# The stoneflies (Insecta, Plecoptera) of the Talladega Mountain region, Alabama, USA: distribution, elevation, endemism, and rarity patterns

**DOI:** 10.3897/BDJ.6.e22839

**Published:** 2018-02-01

**Authors:** Scott A Grubbs, Andrew L Sheldon

**Affiliations:** 1 Western Kentucky University, Department of Biology and Center for Biodiversity Studies, Bowling Green, United States of America; 2 Division of Biological Sciences, University of Montana, Missoula, United States of America; 3 Crawfordsville, Florida, United States of America

**Keywords:** Alabama, Talladega Mountains, Plecoptera, stoneflies, distribution, endemism

## Abstract

Background

The Talladega Mountain region of eastern Alabama is the southernmost outlier of the ancient Appalachian Mountains, including the highest peaks and ranges in the state. Collections of stoneflies (Plecoptera) previously here have been sporadic yet has led to several new species descriptions in modern times (James 1974, James 1976, Stark and Szczytko 1976, Kondratieff and Kirchner 1996, Szczytko and Kondratieff 2015) and expanded our understanding of southeastern US stoneflies. During the period 2003–2012 we conducted an intensive inventory of the stonefly fauna of the Talladega Mountain region. We collected across all months from 192 unique localities, covering a broad range of stream sizes and elevation gradients present in the region.

New information

A total of 57 confirmed species across eight of the nine Nearctic families were collected as adults (Table 4), including four species described as new during the study period (Table 2). *Leuctra crossi* James, 1974 was easily the most common species collected. Median elevations per species ranged from 174 m (*Clioperla clio* (Newman, 1839)) to 410 m (*Leuctra triloba* Claassen, 1923 (Fig. 3). Dot distribution maps were included for all 57 species plus one for undetermined nymphs of *Pteronarcys* Newman, 1838 (Figs. 4–19). As many as seven species may be endemic to the region but sampling efforts northeastward into Georgia, plus additional focused sampling in Alabama and a comprehensive examination of all available material held in museums and personal collections, are needed for confirmation.

## Introduction

Landscapes and riverscapes continue to be altered by anthropogenic activities (Sisk et al. 1994). The literature is replete with examples of how our actions have led to reductions in species richness and genetic diversity. Notable examples include the influences of agriculture (e.g. Poole and Downing 2004, Beketov et al. 2013), climate change (e.g. Buisson et al. 2008, Jordan et al. 2016), deforestation (e.g. Benstead et al. 2003, Sweeney et al. 2004), exotic and invasive species (Mooney and Cleland 2001, Rahel et al. 2008), mining (e.g. Palmer et al. 2010, Hogsden and Harding 2011), reservoir construction and fluvial discontinuity (e.g. Bednarek 2001; Matthews and Marsh-Matthews 2007), and urbanization (e.g. Morgan and Cushman 2005, Nelson et al. 2009).

Stoneflies are aquatic insects that are sensitive indicators of habitat and water quality conditions (Stewart and Stark 2002). Master et al. (2000) listed stoneflies as the third most imperiled biotic group across aquatic and terrestrial systems in the United States. There is ample evidence that several midwestern USA states have experienced extirpation of their native stonefly fauna since the 1950s. For example, 18 (25% of the state’s native fauna) and 10 (12%) species are considered extirpated from Illinois and Indiana, respectively (DeWalt and Grubbs 2011). Extirpation is not unique to North America. In Europe, large river species have disappeared due to organic enrichment and decreases in dissolved oxygen levels (Zwick 1992,Bojkova et al. 2012).

Climate change is affecting biological systems globally both in aquatic and terrestrial habitats (Walther 2010). Mountain ranges are particularly vulnerable due to the influence of “summit traps”, especially in low-elevation montane systems (Sauer et al. 2011, Sheldon and Grubbs 2014). Stoneflies include several cold-stenothermal taxa (Zwick 2000) whose distributions are restricted to montane, high latitude, or spring-fed systems. Consequently, cold stenotherms are considered highly vulnerable to climate warming (Tierno de Figueroa et al. 2010). For example, two stoneflies endemic to the Waterton-Glacier International Peace Park (Alberta, Canada and Montana, USA), *Lednia tumana* (Ricker, 1952) and *Zapada glacier* (Baumann and Gaufin, 1971), have experienced reductions in range and genetic diversity with concomitant loss of glaciers and snowfields (Giersch et al. 2015, Jordan et al. 2016).

Natural areas (e.g. nature reserves, national parks) are widespread throughout the US and have the potential to conserve ecosystems and native fauna and flora (Jenkins et al. 2015). Unfortunately, the task of providing biotic protection is not always realized (Venter et al. 2014) despite the fact that distributional data is increasingly more accessible to land managers (Pimm et al. 2014). Focused surveys and monitoring programs have the capacity to increase our understanding of how protected areas support and protect regional species pools.

Our overall objective of this study was to thoroughly inventory the stonefly fauna of a significant focal area, the Talladega Mountain region (eastern Alabama, USA), by collecting across months and years from multiple localities representative of the broad range of stream sizes and elevation gradients. We intended these data to useful in conservation and land management applications, adequate for ecological, systematic and biogeographic analyses, and a firm basis for designing subsequent research on the ecology of stoneflies of this interesting region.

## What is the Talladega Mountain region?

**Figure 1.**
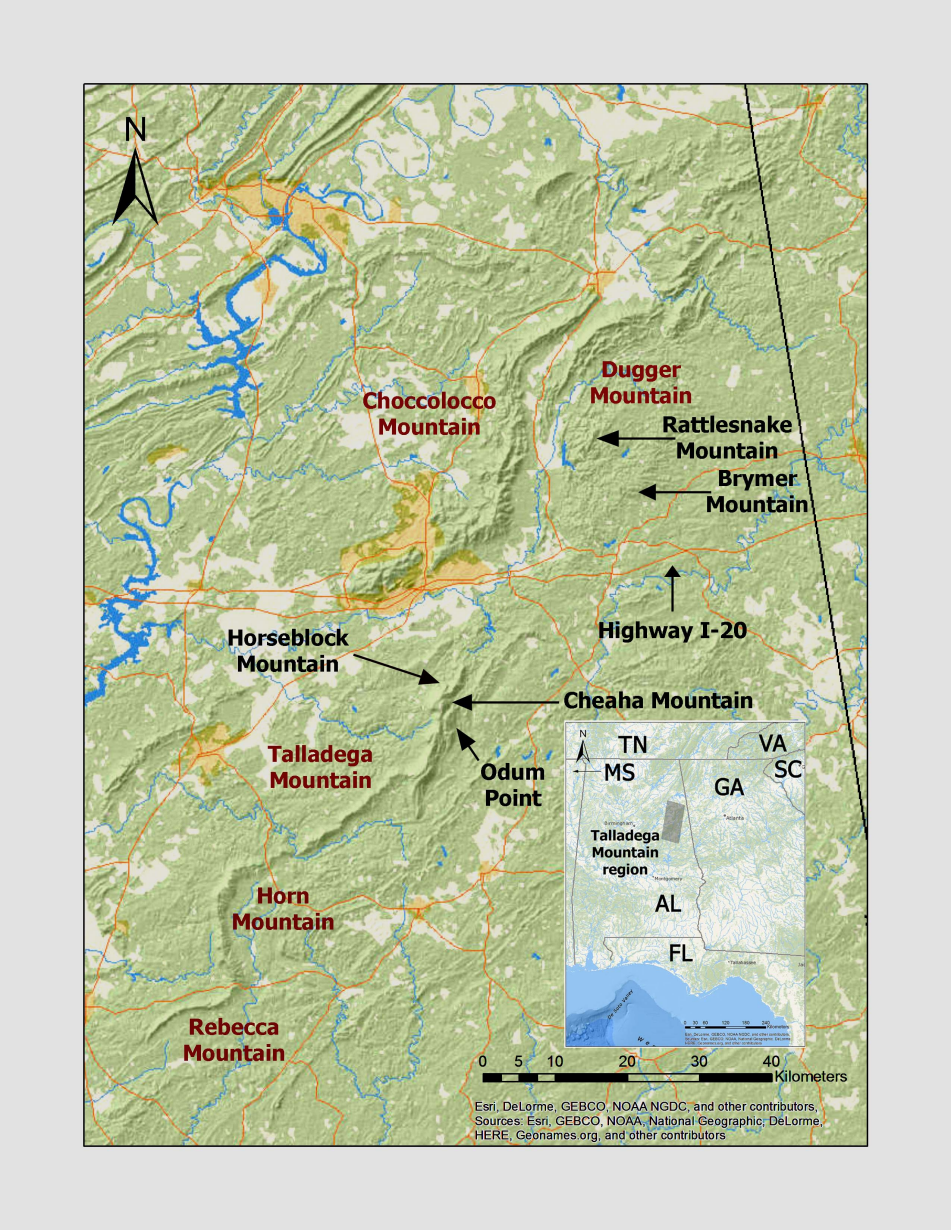
General outline of the Talladega Mountain region in the southeastern USA (shaded box in inset) and prominent peaks and ridges. Peaks are noted by arrows and black type and ridges are noted by magenta type. AL = Alabama, FL = Florida, GA = Georgia, MS = Mississippi, SC = South Carolina, TN = Tennessee, VA = Virginia.

The Talladega Mountain region, as the southernmost outlier of the ancient Appalachian Mountains, represents a unique location in the biological and landscape diversity of the southeastern USA (Duncan 2013). This region consists of metamorphic and igneous rocks and is a composite of several low-lying ridges and other uplifted regions and adjacent valleys in the Piedmont plus Ridge and Valley Physiographic Provinces in eastern Alabama (Fig. 1). For convenience, The Talladega Mountain region can be dissected into “southern” and “northern” sections by Highway Interstate 20 (I-20; Fig. 1).

Starting southward in the Piedmont Physiographic Province, Talladega Mountain in a broad sense is a composite of several long and narrow ridges that include Rebecca Mountain, Horn Mountain, and Cheaha Mountain (= high point in Alabama, 734 m/2407 ft), plus several other high peaks (e.g. Odum Point, 714 m/2342 ft) within the region (Fig. 1). These ridges are defined by erosion resistant metamorphic rock (Cheaha Quartzite) and bordered by terrain of moderate relief on softer Lay Dam Formation (Geological Survey of Alabama 1988). The exposed quartzite ends beyond Cheaha Mountain and the mountainous region continue north of Highway I-20 as an uplifted region that includes Brymer Mountain (442 m/1450 ft) and Rattlesnake Mountain (509 m/1670 ft). The region contacts sedimentary rocks of the Valley and Ridge Province northward along County Route 55. The dominant formation in the northern portion of the region is the Weisner Ridge Sandstone and some Shady Dolomite, reaching 652 m (2140 ft) at Dugger Mountain. Located immediately west and extending southward towards Highway I-20 is Choccolocco Mountain, the other prominent Ridge and Valley structure in this region. Choccolocco Mountain peaks at 629 m (2063 ft) and is also part of the Weisner Ridges with outcroppings of Weisner Quartzite.

The regional climate is highly variable and characterized by hot summers, cool winters, and moderate rainfall. The period extending from August–October is a dry season. Heflin, AL (259 m) has January and July mean temperatures of 4.9 °C and 25.6 °C, respectively, and receives ca. 140 cm/yr precipitation (National Oceanic and Atmospheric Administration 2017). During our study (2003–2012) the annual minima were -14.4 – -8.9 °C and maxima 32.8–41.1 °C. Snowfall is infrequent but we encountered some snow during field work at higher elevations. In the drought year of 2007 (Maxwell and Soulé 2009), Heflin received only 69 cm of precipitation and some normally perennial streams were dry. In contrast, Heflin received 191 cm of precipitation during a wet year in 2009.

**Figure 2.**
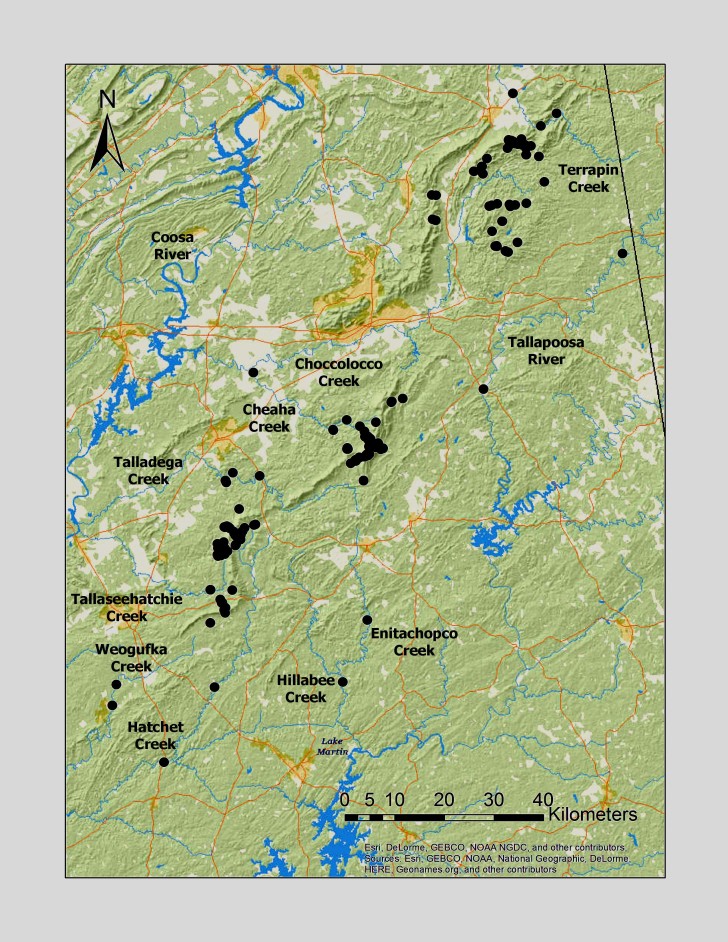
All unique collection localities (n = 192) in this study and the locations of the larger streams and rivers in the region.

Streams are numerous and the entire region is nested within the Coosa River Basin. Upland streams drain northward to Terrapin Creek, eastward into the Tallapoosa River, southward into Lake Martin, or westward directly to the Coosa River (Fig. 2). Most upland streams are unregulated but three impoundments influence upper Shoal Creek and there is a small impoundment in the Dry Creek headwaters within Cheaha State Park. Larger regional streams support diverse assemblages of fishes, mussels, and snails, including several species of conservation concern (Boschung and O'Neil 1981, Mirarchi et al. 2004, Meade et al. 2009, Edelman et al. 2015).

Forest composition varies from diverse bottomland hardwood forests, including some loblolly pine (*Pinus taeda* L.), to pine-dominated (shortleaf pine *P. echinata* Mill., longleaf pine *P. palustris* Mill., and Virginia pine *P. virginiana* Mill.) forests with several species of oak (*Quercus* L.) at higher elevations and on drier sites (Shankman and Wills Jr. 1995, Womack and Carter 2011). Establishment of loblolly pine plantations and restoration of longleaf pine savannas (Duncan 2013) continue to alter the forest landscape. Much of the range is publically owned and managed by the Talladega National Forest (Talladega and Shoal Creek Ranger Districts) and Cheaha State Park. When the Talladega National Forest was established in 1936 about 30% of its area lacked forest (Duncan 2013). Forest cover, including pine plantations and successional stages, is virtually complete at present.

## Stonefly Collections in the Talladega Mountain region

Collections of stoneflies (Plecoptera) previously here have been sporadic yet has led to several new species descriptions in modern times (James 1974, James 1976, Stark and Szczytko 1976, Kondratieff and Kirchner 1996, Szczytko and Kondratieff 2015). The first records of stoneflies from the Talladega Mountain region were given by Audrey James in her Ph.D research on Alabama Plecoptera (James 1972). Although she did not use the words “Talladega Mountains” in her dissertation, dot distribution maps (her Figs. 111–136) depicted 32 species from the region at that time. Four new regional species were included as informal manuscript names that would be formally described shortly thereafter in James (1974, 1976; Table 1). Stark and Harris (1986) made several references to stonefly species from the Talladega region but only by county (i.e. Calhoun, Cleburne, Clay, and Talladega). Feminella (1996) compared aquatic insect communities along flow permanence gradients in six Talladega National Forest streams, but all stonefly taxa reported were determined only to the generic level. Since James (1974, 1976), seven species have been described from the Talladega Mountain region (Table 2).

The origin of a biogeographic study of the stonefly fauna of the Talladega Mountains occurred after SAG traveled to the region in February 2003 looking extensively for the nemourid genus *Soyedina* Ricker, 1952 (none were ever found). Collections during that first trip, however, provided material leading to the description of *Zealeuctra talladega* Grubbs, 2005 and the first male specimen of *Allocapnia menawa* Grubbs and Sheldon, 2008. Shortly thereafter, ALS contacted SAG about collaborating on a focused research project and a formal sampling concept was conceived and commenced in 2005. ALS has collected mainly in upland streams throughout the region whereas SAG provided complimentary work both in upland streams and from the largest streams draining northward and southward. In total, 26 collecting trips occurred as independent endeavors by the authors between 2003 and 2012 (Table 3).

## Field Methods

**Figure 3.**
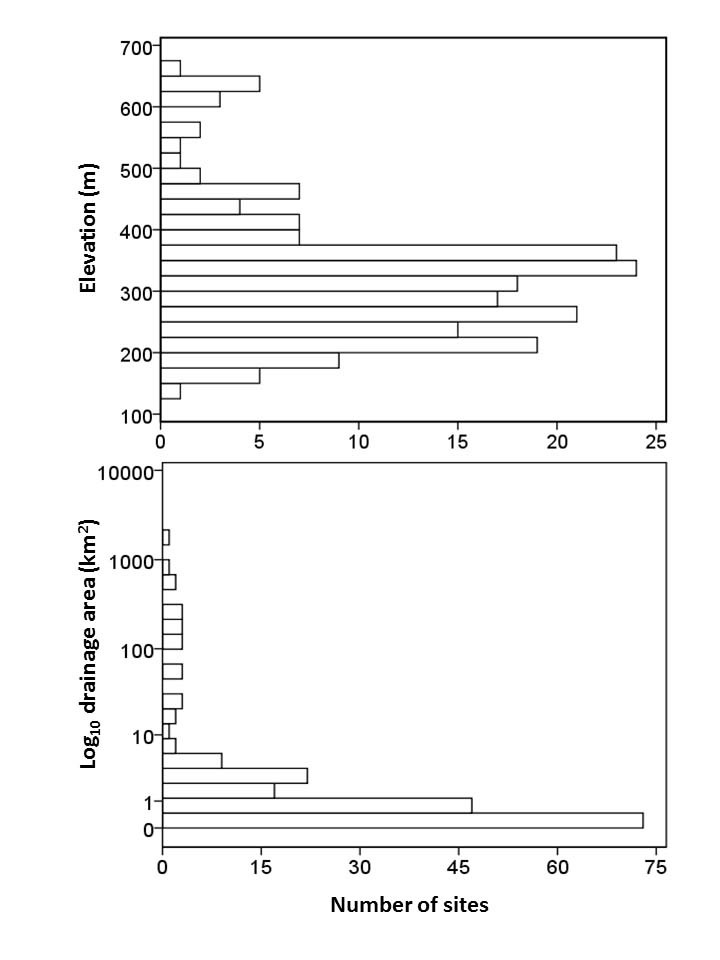
Frequency distribution plots of unique collecting sites by elevation (m) and drainage area (km^2^).

Most sampling in upland streams occurred in Talladega National Forest at U.S. Forest Service road crossings, adjacent to campgrounds (e.g. Turnipseed), along established hiking trails (e.g. Pinhoti National Recreation Trail), and by hiking off trail along streams. Larger streams (e.g. Hatchet Creek) were located mainly at road crossings adjacent to private land. In total, we have positive collections from 192 unique sites (Fig. 2). Some sites were located at different elevations along the same stream (e.g. Swept Creek). Sites ranged in elevation from 131–660 m (429–2165 ft). Most sites, however, were nested within a narrow range of elevations (190–390 m, n = 150) and basin areas (< 25 km^2^, n = 176) (Fig. 3). The least common streams available for collecting were those at higher (> 500 m, n = 13) and lower (< 190 m, n = 9) elevations, and in basin areas larger than 100 km^2^ (n = 13) (Fig. 3).

Nearly all data (ca. 99%) presented in this treatment were based on adult specimens. Adults, particularly males, provide the best and most objective set of characteristics for identifying species. This paper does not include the nymphal data of *Acroneuria abnormis* (Newman, 1838), *Beloneuria jamesae* Stark and Szczytko, 1976, and *Eccoptura xanthenes* (Newman, 1838) presented in Sheldon and Grubbs (2014). Adults were collected with a beating sheet to dislodge specimens from riparian vegetation, hand-picking from rocks, leaf packs, tree trunks and bridges, and use of light traps on warm evenings. The latter is especially effective for members of the family Perlidae. Specimens were preserved on site in 75–95% ethanol.

## Laboratory Methods and Data Management

Location data (in decimal degrees) for each specimen record were recorded either directly on site with a portable GPS unit or georeferenced from vial label data using Acme Mapper 2016 (https://mapper.acme.com/; datum WGS-84). Drainage area for each sampling location was determined using the CONTDA function in StreamStats 4.0 (https://streamstatsags.cr.usgs.gov/streamstats). We used drainage area as a proxy of stream size.

Regional distribution maps for all species were initially prepared using an ArcGIS public web account (http://www.arcgis.com/home) and then finished using ArcMap 10.2. Latitude and longitude coordinates in decimal degrees of all collection records for each species were overlaid on a public domain map titled “Oceans”.

With the exception of type material for the four species previously described as new during this study (Table 2), most material is currently stored in 75–95% ethanol at Western Kentucky University (WKUC). All materials collected by ALS were referenced by a unique field number identifier. Data are presented in two formats. Collections are organized chronologically (Table 3) following the recommendation of Sheldon (2016) that multispecies collection data are primary, informative, and readily available. Species records, also organized chronologically, are archived in Darwin Core Archive file format supported by Pensoft's Integrated Publishing Toolkit and posted at the Global Biodiversity Information Facility (GBIF) (Grubbs and Sheldon 2017).

## Results and Discussion

### Species present and comparisons to prior research

Nearly 700 specimen records (= vials) and > 3200 individual specimens were obtained during this study, resulting in 57 verified species (Table 4). This species tally is conservative since we did not collect *Perlinella drymo* (Newman, 1839), adults of *Pteronarcys* Newman, 1838, and other species we expected to find during this study (see below). For several reasons outlined below, seven species listed from the region by James (1972) are not represented in Table 4.

The most speciose families found in the region as adults were Perlidae (n = 12 species), followed by Leuctridae and Perlodidae (n = 10 species each), and Capniidae (n = 9 species) (Table 4). *Leuctra* Stephens, 1836 (n = 9 species), *Allocapnia* Claassen, 1928 (n = 8 species), and *Isoperla* Banks, 1906 (n = 6 species) were the most speciose genera present (Table 4). Although all nine Nearctic families are present in the region, we did not collect adult specimens of *Pteronarcys*. The Alabama state record of *P. biloba* Newman, 1838 that arose from a single nymphal specimen from Cleburne County in James (1972, her Fig. 127), and carried forward in Stark and Harris (1986), Grubbs (2011), and DeWalt et al. (2017), is still considered as tentative since this specimen has not been located for study. Similarly, ALS collected *Pteronarcys* nymphs from eight streams (Sheldon and Grubbs 2014) that have been tentatively determined as *P. biloba*. We have also collected nymphs lacking abdominal spines that are either *P. dorsata* (Say, 1823) or *P. pictetii* (Hagen, 1873). *Pteronarcys* nymphs can problematic to identify to species, however, so adults are needed for confirmation. A regional distribution map for *Pteronarcys* spp. nymphs was included in hopes that adults of both nymphal types (i.e. abdomen with and lacking lateral spines) will be collected (Fig. 19)

Four plotted records of *Leuctra* presented in James (1972) require clarification (Table 1). First, she plotted single records of *L. alabama* James, 1974 and *L. alexanderi* Hanson, 1941 from Calhoun and Jackson counties, respectively (her Fig. 115). These distributional points may have been inadvertently switched. *Leuctra alabama* was later formally described (James 1974) from a single locality in Jackson County (far northeastern Alabama) that appears to match identically to Fig. 115 in James (1972). Her singular record of *L. alexanderi* refers to the Calhoun County type locality of *L. crossi* James, 1976, a species she noted was morphologically similar to *L. alexanderi* in James (1972). Second, her simple line drawing (her Fig. 33) of *L. biloba* Claassen, 1923 is easily interpretted as *L. grandis* Banks, 1906. Grubbs (2010) first reported *L. grandis* from the Talladega Mountains and in this study it was commonly collected across the region (Table 4). The dorsal abdominal lobes of *L. grandis* are consistently triangular across the broad range of this widespread Appalachian species, which is in sharp contrast to the bluntly rectangular lobes exhibited by *L. biloba* (Grubbs, unpublished research). Third, we did not collect *L. moha* Ricker, 1952 during this study. James (1972) reported this species from Alabama based only on females, including a regional locality from southern Clay County. All four regional species of *Leuctra* with autumnal emergence periods (*L. cottaquilla* James, 1974, *L. ferruginea* (Walker, 1852), *L. tenuis* (Pictet, 1841), *L. triloba* Claassen, 1923) were not collected during September–November south of streams draining Cheaha Mountain and Odum Point (Fig. 1). *Leuctra triloba* was collected from Horn Mountain but only in late January. Harrison and Stark (2010) suggested deletion of *L. moha* from the Alabama state list. The morphology and distribution of *L. moha* is poorly understood with confirmed records to date only from Georgia and South Carolina (DeWalt et al. 2017).

We collected two species of *Neoperla* Needham, 1905 and four species of *Perlesta* Banks, 1906 during this study (Table 4). At the time of James (1972), however, *Neoperla* was recognized only as a single, variable species (= *N. clymene* (Newman, 1839); Stark and Baumann 1978). The regional site record plotted in James (1972, her Fig. 128) could be one of several species, including the two reported here (Table 4). Similarly, in the early 1970s *Perlesta* was recognized as *P. placida* (Hagen, 1861), a species also of variable form at that time, and *P. frisoni* Banks, 1948 (Stark 1989). The multiple regional records of *P. placida* plotted in James (1972, her Fig. 131) could have referred to any of the four species reported here (Table 4), including the true *P. placida*. In addition, we did not collect *Perlinella drymo* during this study. James (1972, her Fig. 131) plotted a single regional record for *P. drymo* from Cleburne County.

The absence of two subfamilies from the region that include eastern Nearctic species needs mention. First, the genus *Megaleuctra* Neave, 1934 (Leuctridae: Megaleuctrinae) does not extend southward into the Talladega Mountains. The two eastern Nearctic *Megaleuctra* species are restricted to cold-water systems located farther north (Baumann and Stark 2013). ALS made numerous attempts to collect *Megaleuctra* from high elevation seeps and springs but was unsuccessful. Second, we did not collect non-gilled nemourids (Nemouridae: Nemourinae) in the region. In addition to *Soyedina*, we had anticipated that species of two additional genera were present in the region. *Ostrocerca truncata* (Claassen, 1923) can be a locally abundant species in headwater streams, is distributed extensively along the Appalachian Mountains (DeWalt et al. 2017), and has been reported <100 km northward in DeSoto State Park (DeKalb County; Young et al. 1989). A second species, *Prostoia completa* (Walker, 1852), is typically found in small rivers and large streams through eastern and central North America (Grubbs et al. 2014) and was plotted in James (1972, her Fig. 126) from southern Tallapoosa County.

### Elevation trends and common vs. uncommon regional species

**Figure 4.**
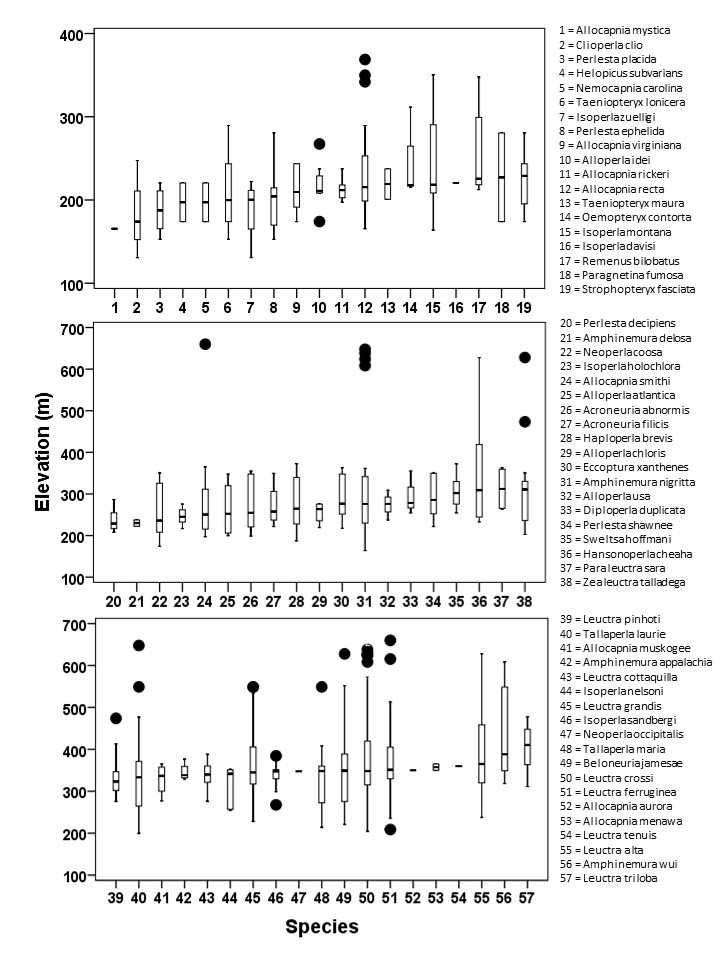
Box-and-whisker plots arranging species by median elevation at unique sites from low (top) to high (bottom). Round black symbols represent outliers not used to calculate median values (horizontal black bar), interquartile range (box), or whiskers (range).

Median elevations per species ranged from 174 m (*Clioperla clio* (Newman, 1839)) to 410 m (*Leuctra triloba*) (Fig. 4). A single female of *C. clio* was collected from the lowest elevation stream (Hatchet Creek, 131 m) whereas *Allocapnia smithi* Ross and Ricker, 1971 and *Leuctra ferruginea* were collected from springs draining into the upper reaches of Talladega Creek at 660 m. Distinct elevation trends were seen within individual families. All regional species of Leuctridae were found mainly in upland, higher elevation streams (Fig. 4): *Leuctra alta* James, 1974 (Fig. 7; median = 358 m), *L. cottaquilla* (Fig. 7; median = 340 m), *L. crossi* (Fig. 7; median = 348 m), *L. ferruginea* (Fig. 8; median = 351 m), *L. grandis* (Fig. 8; median = 348 m), *L. pinhoti* Grubbs and Sheldon, 2009 (Fig. 8; median = 323 m), *L. tenuis* (Fig. 8; 360 m), *L. triloba* (Fig. 9; median = 410 m), *Paraleuctra sara* (Claassen, 1937) (Fig. 9; median = 312 m), and *Zealeuctra talladega* (Fig. 9; median = 311 m). Both regional species of Peltoperlidae were found commonly in upland, higher elevation streams (Fig. 4): *Tallaperla laurie* (Ricker, 1952) (Fig. 13, median = 334 m) and *T. maria* (Needham and Smith, 1916) (Fig. 13, median = 348 m). With the exception of *Alloperla idei* (Ricker, 1935), most species of Chloroperlidae were also characteristic of upland streams (Fig. 4): *A. atlantica* Baumann, 1974 (Fig. 11, median = 250 m), *A. chloris* Frison, 1934 (Fig. 12, median = 276 m), *A. usa* Ricker, 1952 (Fig. 12, median = 276 m), *Haploperla brevis* (Banks, 1895) Fig. 12, median = 264 m), and *Sweltsa hoffmani* Kondratieff and Kirchner, 2009 (Fig. 13, median = 302 m).

In contrast, all four regional species of Taeniopterygidae were found in low elevation streams (Fig. 4): *Oemopteryx contorta* (Needham and Claassen, 1925) (Fig. 10; median = 218 m), *Strophopteryx fasciata* (Burmeister, 1839) (Fig. 11; median = 229 m), *Taeniopteryx lonicera* Ricker and Ross, 1968 (Fig. 11; median = 201 m), and *T. maura* (Pictet, 1841) (Fig. 11; median = 201 m). Three regional species of the perlid genus *Perlesta* were also found in low elevation systems (Fig. 4): *P. decipiens* (Walsh, 1862) (Fig. 15; median = 229 m), *P. ephelida* Grubbs and DeWalt, 2012 (Fig. 16; median = 204 m), and *P. placida* (Fig. 16; median = 188 m). *Perlesta shawnee* Grubbs, 2005, however, was typically found in smaller, upland streams (Fig. 16; median = 286 m). The two large-bodied perlid species that appear to be regional endemics, *Beloneuria jamesae* (Fig. 14, median = 349 m) and *Hansonoperla cheaha* (Fig. 14, median = 309 m), are also characteristic of small, upland streams (Sheldon and Grubbs 2014).

Increasing altitude with concomitant reduction in flow permanence and stream size, however, had no influence on uncommon or rarity patterns. Although we found 12 species at higher elevation sites (> 500 m), all were collected commonly during this study (range: 8–61 unique localities/species). *Leuctra crossi* was also the most common species found at higher elevation sites (n = 8 unique sites > 500 m), followed by *Amphinemura nigritta* (Provancher, 1876) (n = 4 unique sites), and *L. alta*, *L. ferruginea*, and *A. wui* (Claassen, 1936) (n = 3 unique sites each).

Overall the most common regional species was *Leuctra crossi*, collected at 61 unique localities (Fig. 7, Table 4). This was > 15 localities compared to the five next most common species: *Amphinemura nigritta* (Fig. 10, n = 43), *Tallaperla laurie* (Fig. 13, n = 38), *Haploperla brevis* (Fig. 12, n = 37), *T. maria* (Fig. 13, n = 31), and *L. grandis* (Fig. 8, n = 28) (Table 4). Overall, 20 species were collected from ≥ 10 unique localities.

Eleven species (= 19% of regional fauna) were obtained at only one or two unique localities (Table 4). *Allocapnia mystica* Frison, 1929 (Fig. 5), *Nemocapnia carolina* Banks, 1938 (Fig. 7), *Amphinemura delosa* (Ricker, 1952) (Fig. 10), *Taeniopteryx maura* (Fig. 11), *Paragnetina fumosa* (Banks, 1902) (Fig. 15), *Isoperla davisi* James, 1974 (Fig. 17), and *Helopicus subvarians* (Banks, 1920) (Fig. 17) were mainly found at lower elevation sites (range: 166–280 m). Several additional species were also found more commonly (≥ 5 unique sites) from lower elevation sites (Fig. 4). Notable examples include *Allocapnia rickeri* Frison, 1942 (Fig. 6), *Alloperla idei* (Fig. 12), and *Isoperla zuelligi* Szczytko and Kondratieff, 2015 (Fig. 18).

### Succession of adult stoneflies

*Helopicus subvarians* was the only regional species not collected as adults. Adult data is also missing from August (Table 3); this was the only month when neither author travelled to the region. Four species of *Leuctra* were collected during autumn (Table 5), including two species (*L. ferruginea*, *L. triloba*) with extended emergence periods. An extended emergence period for *L. ferruginea* has been shown previously (Harper 1973). The life cycle of *L. triloba* is unknown. Species of the families Capniidae and Taeniopterygidae are “winter stoneflies” (Ross and Ricker 1971) with adult emergence at low elevations and mid-latitudes typically occurring mainly from December through March. All eight regional *Allocapnia* species and all four Taeniopterygidae species were collected as adults between December and March (Table 5). Only *Nemocapnia carolina* was collected in April. *Zealeuctra talladega* was the only other regional species collected through the winter months.

The remaining four regional species of Leuctridae (*L. alta*, *L. crossi*, *L. grandis*, *L. pinhoti*, *P. sara*) and all four species of Nemouridae (*Amphinemura appalachia* Baumann, 1996, *A. delosa*, *A. nigritta*, *A. wui*) commenced emergence during the spring months (Table 5). Although both regional species of *Tallaperla* were often collected together, the emergence period of *T. laurie* extended ca. one month later compared to *T. maria* (Table 5).

Two regional species of Perlodidae, *Clioperla clio* and *Isoperla sandbergi* Szczytko and Kondratieff, 2015, started emergence in late winter (Table 5). The remaining five regional *Isoperla* species were collected as adults only during spring. The two species of Perlodinae collected as adults were found during late spring (*Diploperla duplicata* (Banks, 1920)) or spring-summer (*Remenus bilobatus* (Needham and Claassen, 1925). All six regional Chloroperlidae were also found predominantly in spring (Table 5). *Alloperla chloris* was the only regional chloroperlid species collected into June.

As expected, the family Perlidae was found commonly during the summer months. Although *Perlesta decipiens* and *P. ephelida* were collected by mid-April (Table 5), most perlid species did not emerge until May. Five species, *Acroneuria abnormis*, *A. filicis* Frison, 1942, *Beloneuria jamesae*, *Eccoptura xanthenes*, *Neoperla coosa* Smith and Stark, 1998, and *P. shawnee*, were collected through late July (Table 5) and likely into August had we collected during the latter month.

### Endemics and northern vs. southern regional species

Seven species, including three of the family Leuctridae, may be endemic to the Talladega Mountain region (Table 4). This number should be viewed as tentative, however, because little is understood about statewide distribution patterns of stoneflies across Alabama and northeastward into Georgia. For example, *Allocapnia muskogee* was described as new during this study (Grubbs and Sheldon 2008) but is also known from northern Georgia (Verdone et al. 2017). Except for *Zealeuctra talladega*, all of the supposed endemic species may have distribution patterns extending northward through the Valley and Ridge region into adjacent northwestern Georgia. *Zealeuctra talladega* has been collected only in the southern Talladega Mountain region (Fig. 9). Streams draining Dugger Mountain in the northern portion of the region were particularly emphasized because this is one of the few peaks > 2000 ft (> 610 m) and to provide broad north-south spatial coverage. We have yet, however, to collect *Z. talladega* north of the vicinity of Cheaha Mountain.

Overall, roughly 2/3 (39 species or 68%) of species were collected both north and south of Highway I-20. Several species are likely also more represented along Choccolocco Mountain but much of this area is located in private landholdings and collections by us here were limited both in effort and number (Fig. 2). Several species were found only or mainly in northern or southern portions of the Talladega Mountain region. Characterizing any species as northern or southern Talladega regionals, however, may by spurious. For example, nine species were found only in the southern Talladega region. Yet seven of these species, *Allocapnia smithi* Ross and Ricker, 1971 (Fig. 6), *A. virginiana* Frison, 1942 (Fig. 6), *Nemocapnia carolina* (Fig. 7; Stark et al. 2016), *Amphinemura wui* (Fig. 10), *Oemopteryx contorta* (Fig. 10), *Acroneuria filicis* Frison, 1942 (Fig. 14), and *Helopicus subvarians* (Fig. 17), have distributions farther northward in eastern North America. As indicated above, *Z. talladega* (Fig. 9) appears restricted to the southern Talladega region but there is too little distribution data on *Allocapnia menawa* (Fig. 5) to be confident. On a related note, there are four species that may be restricted to the northern Talladega region: *Leuctra pinhoti* (Fig. 8), *Amphinemura delosa* (Fig. 10), *Taeniopteryx maura* (Fig. 11), and *Alloperla usa* Ricker, 1952 (Fig. 12).

## Conclusions

Our combined efforts across a 10-year period resulted in 57 confirmed species from the Talladega Mountain region. Four new species (Grubbs 2005, Grubbs and Sheldon 2008, Grubbs and Sheldon 2009) were previously described during the study period. We detected clear patterns of stream size and elevation gradients with species abundance data. We expect, however, that more species are present in the region for two reasons. First, we did not collect at least one species previously reported (i.e. *Perlinella drymo*) by James (1972). Second, adults of two species of *Pteronarcys* are present but adults are needed for verification. In addition, there may be other species (i.e. *Ostrocerca truncata*) that we did not obtain yet may be collected at some point in the future. Seven species may be endemic, but focused collecting efforts northeastward into Georgia and examinations of museum holdings and personal collections, are needed for confirmation. This form of baseline data can be useful for managers of national forest lands, especially for species with smaller ranges (i.e. regional endemics) that are of greater risk of extinction (Pimm et al. 2014). Natural areas can protect large proportions of the regional species pool by providing intact habitat and by mitigating development within their boundaries, even in eastern US national forests which are often comprised of a mosaic of public and private landholdings.

## Figures and Tables

**Figure 5a.**
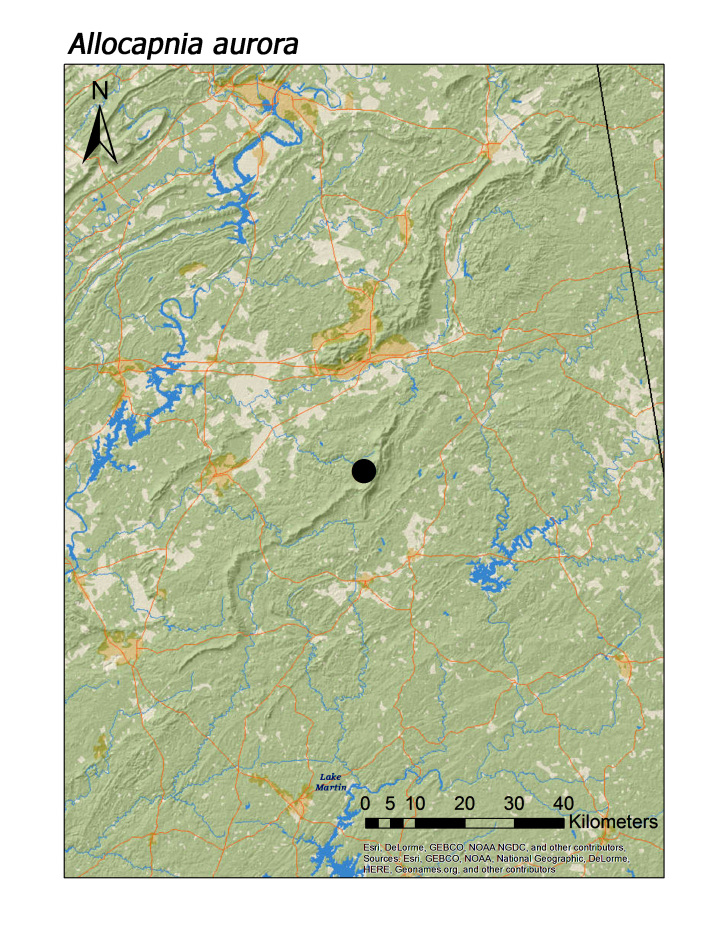
*Allocapnia aurora*

**Figure 5b.**
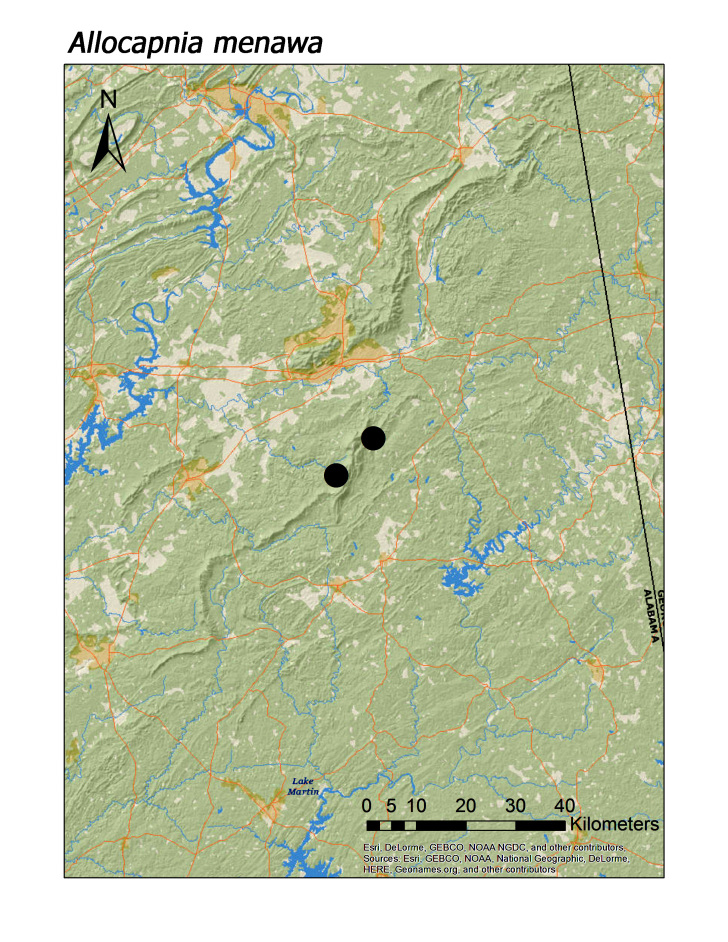
*A. menawa*

**Figure 5c.**
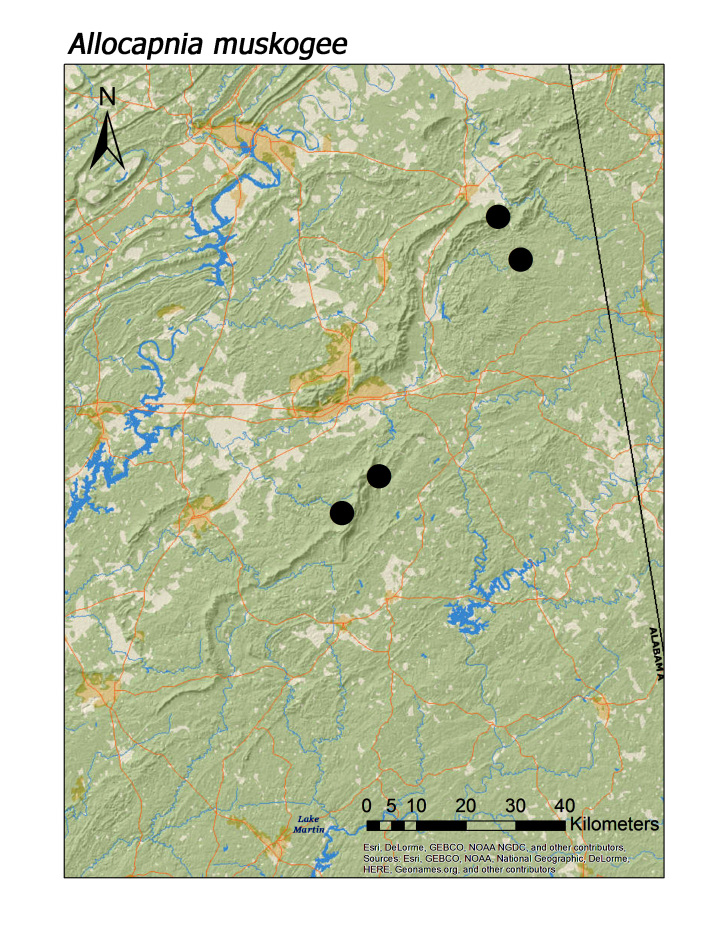
*A. muskogee*

**Figure 5d.**
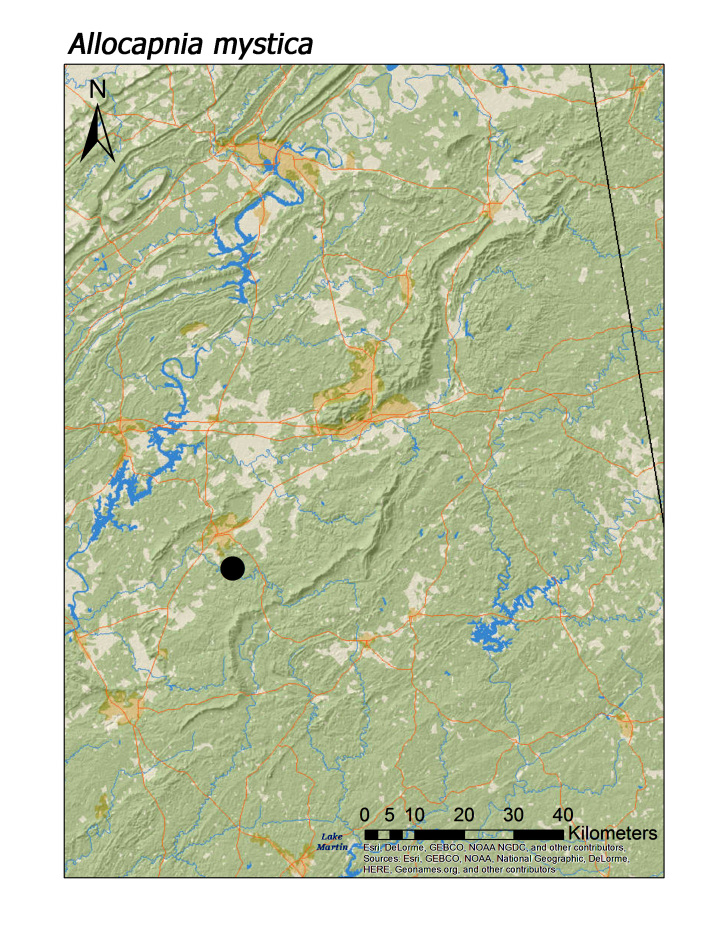
*A. mystica*

**Figure 6a.**
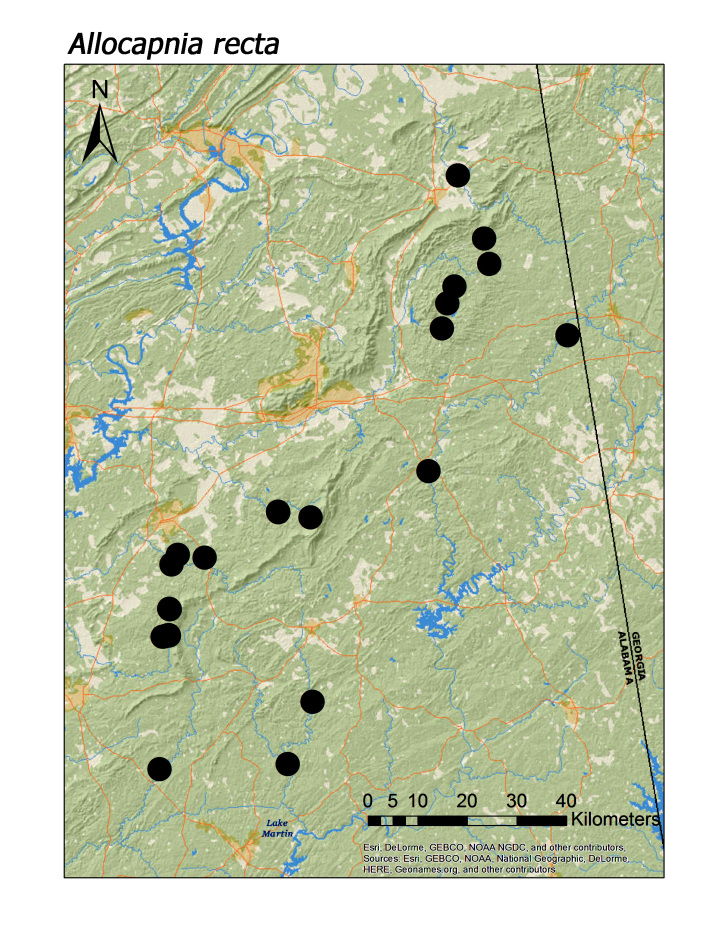
*Allocapnia recta*

**Figure 6b.**
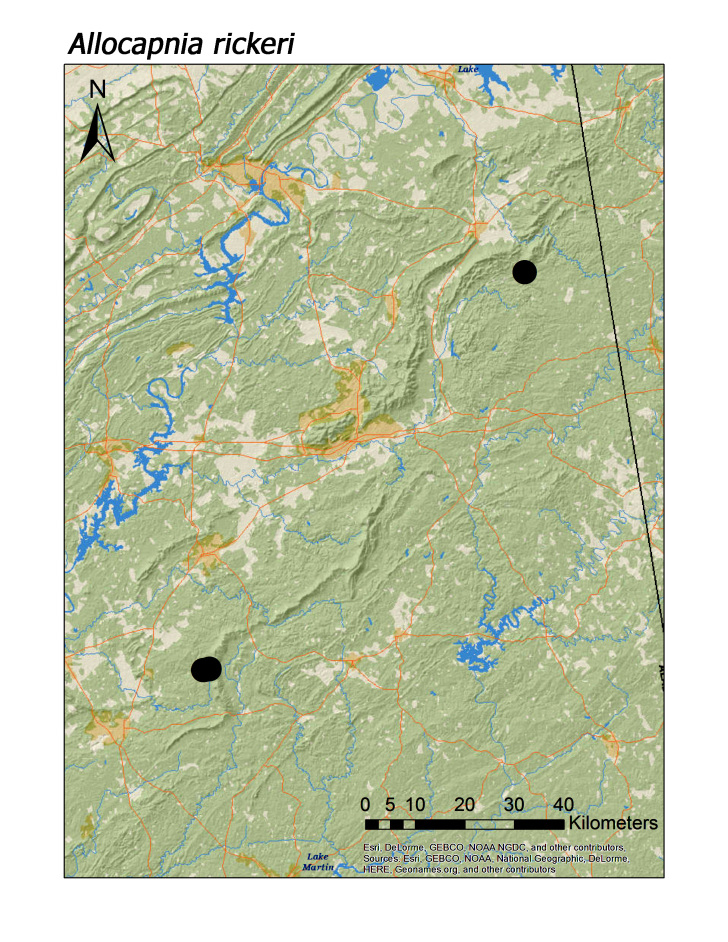
*A. rickeri*

**Figure 6c.**
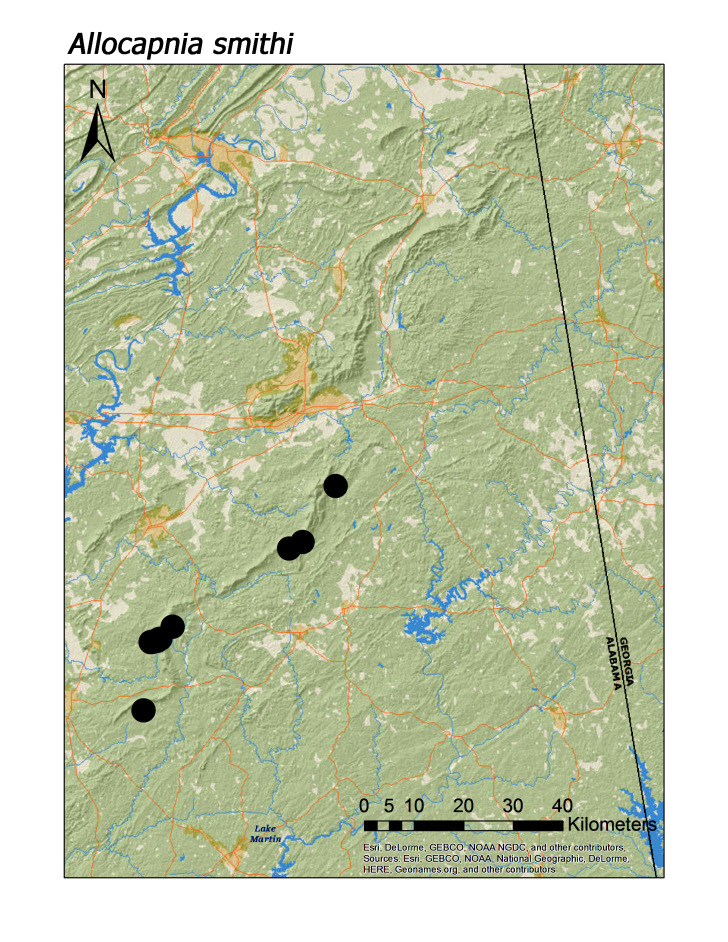
*A. smithi*

**Figure 6d.**
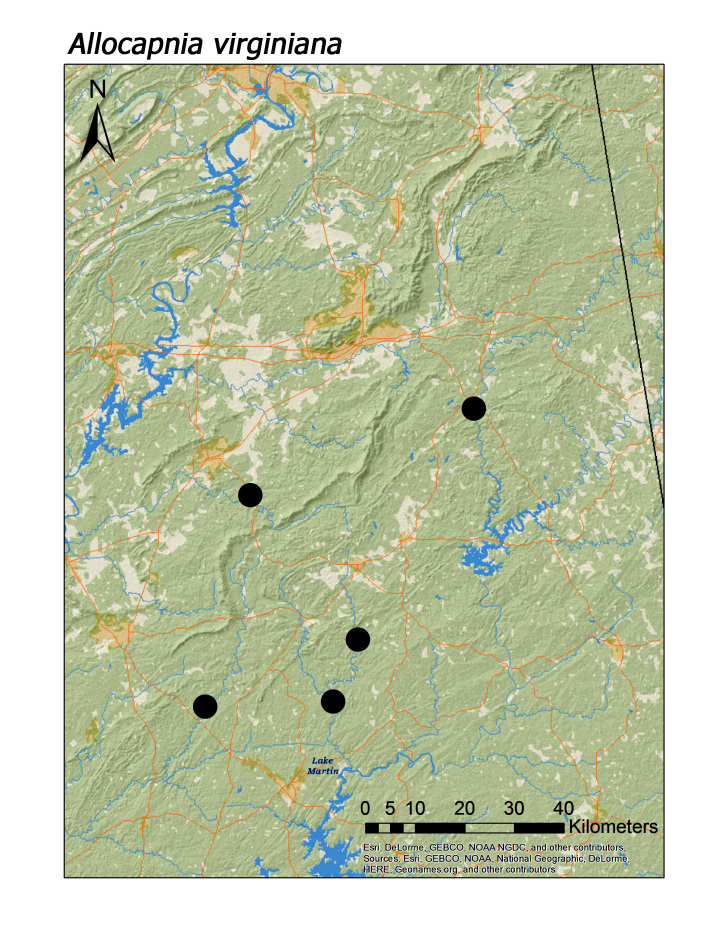
*A. virginiana*

**Figure 7a.**
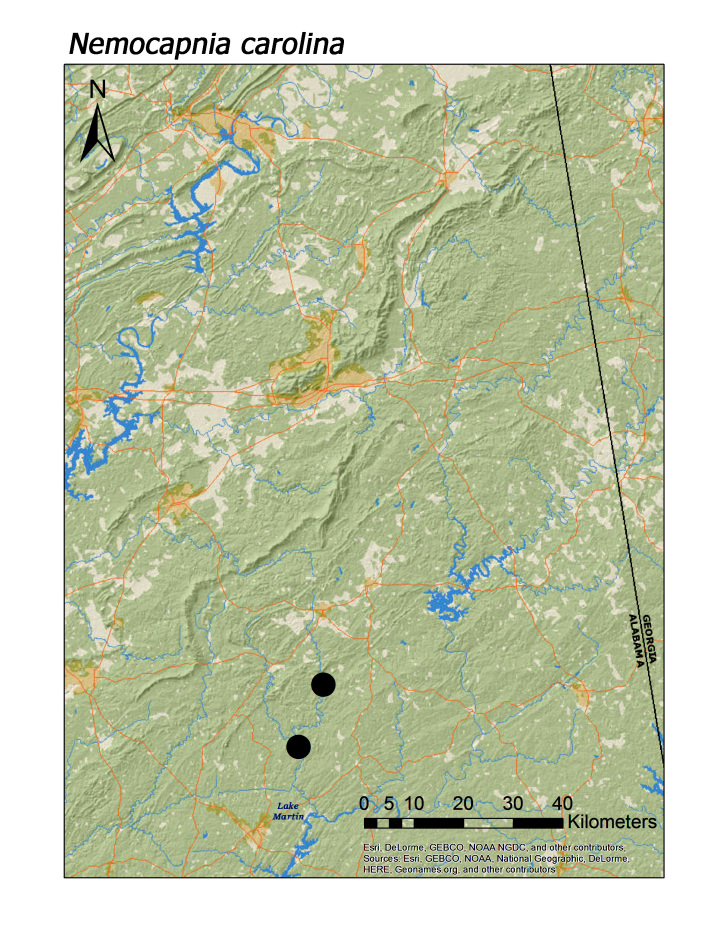
*Nemocapnia carolina*

**Figure 7b.**
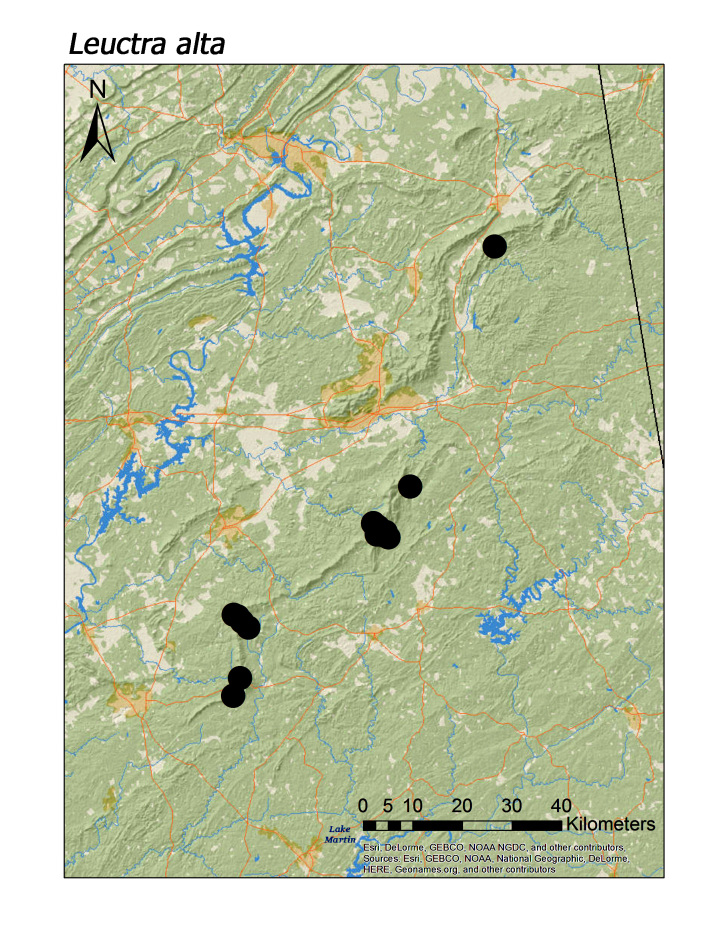
*Leuctra alta*

**Figure 7c.**
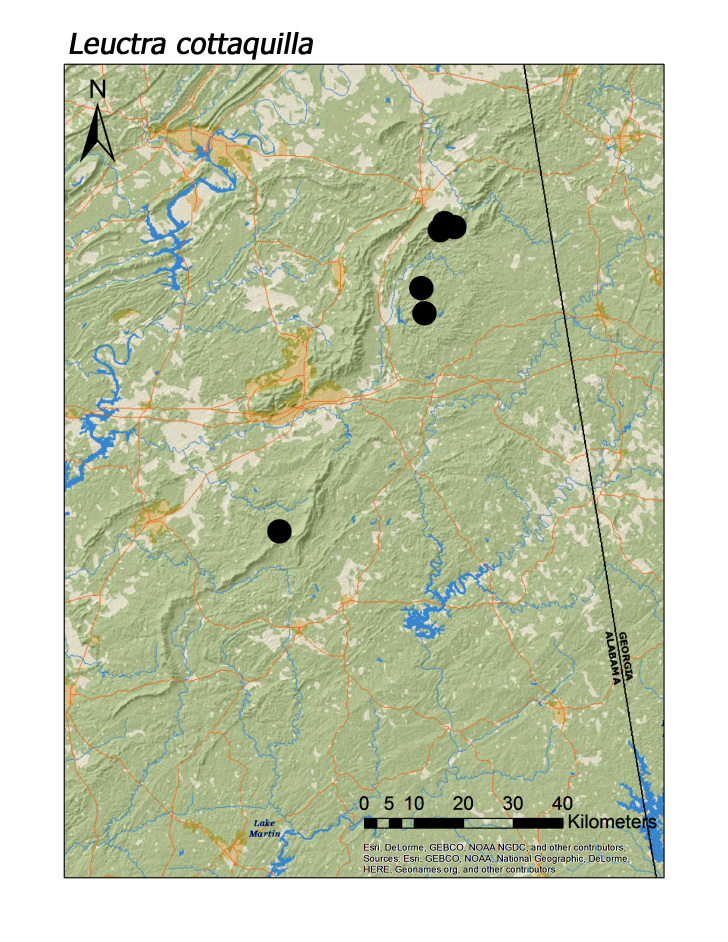
*L. cottaquilla*

**Figure 7d.**
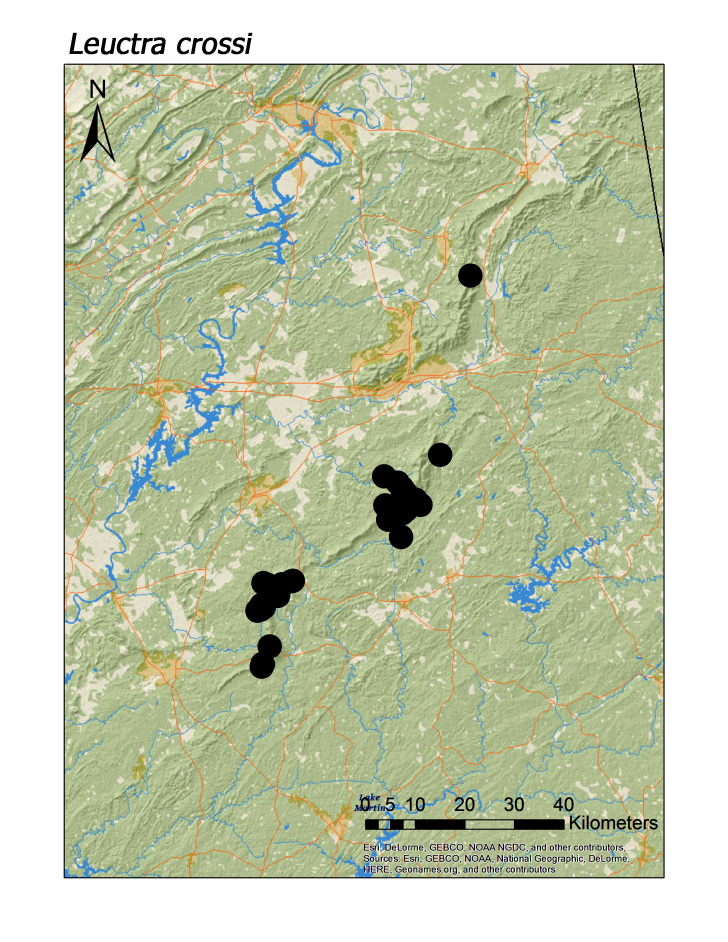
*L. crossi*

**Figure 8a.**
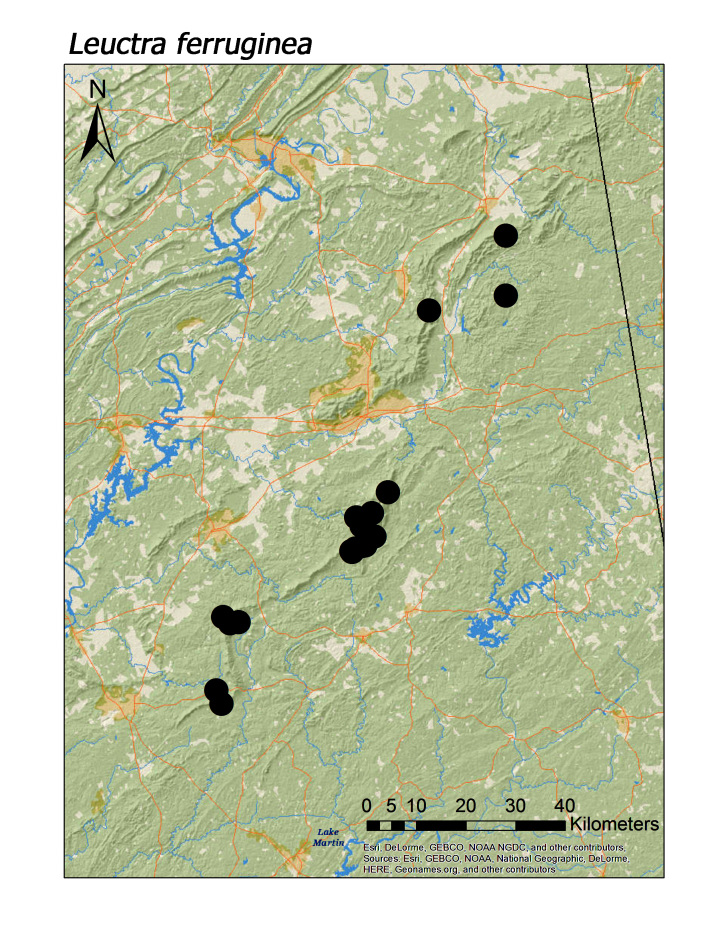
*Leuctra ferruginea*

**Figure 8b.**
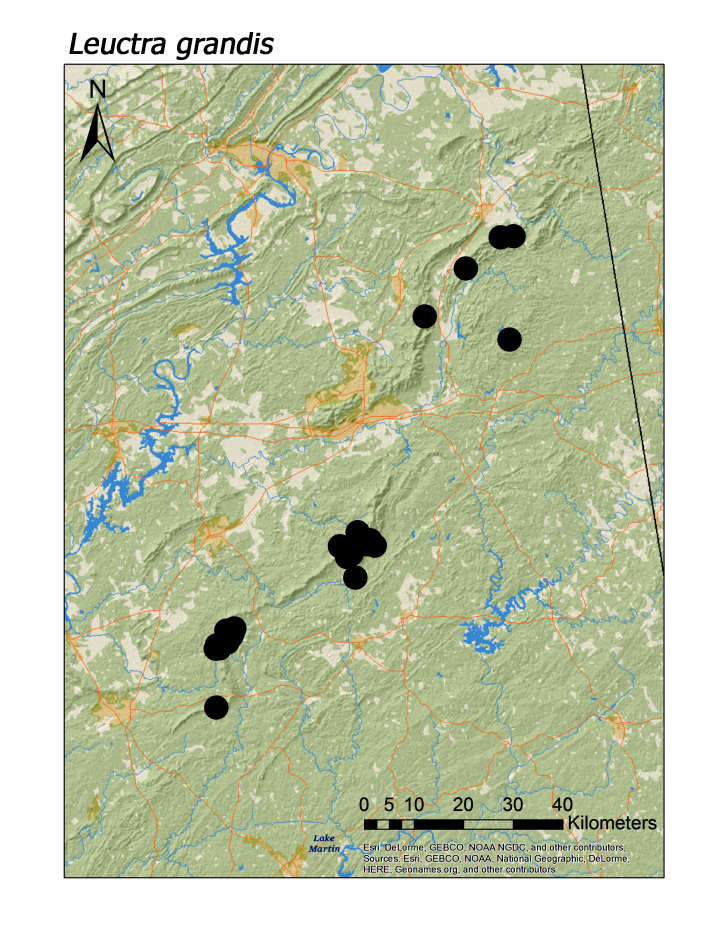
*L. grandis*

**Figure 8c.**
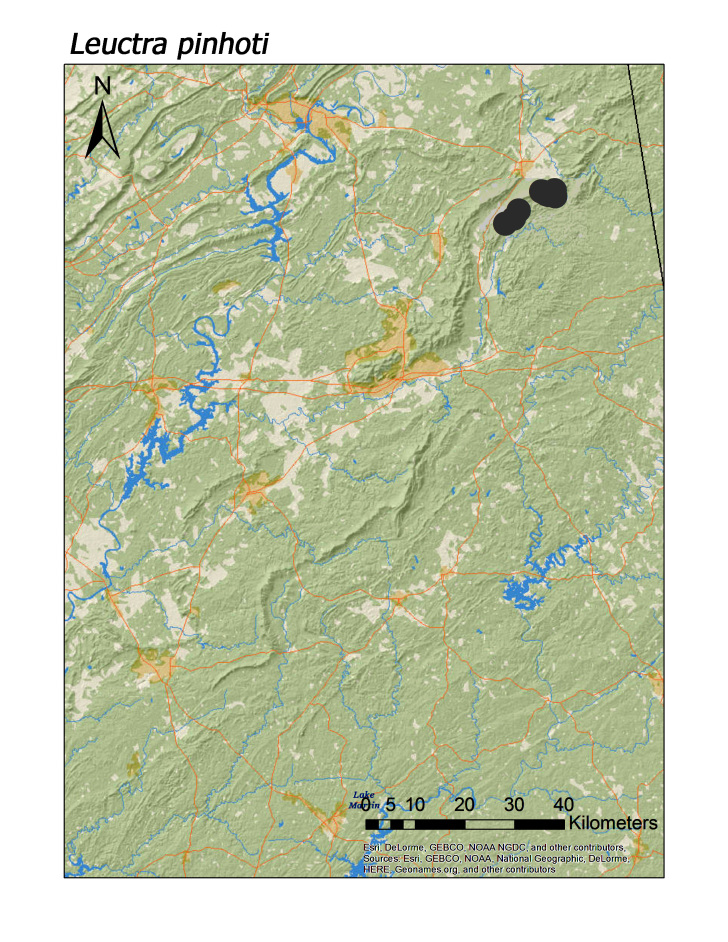
*L. pinhoti*

**Figure 8d.**
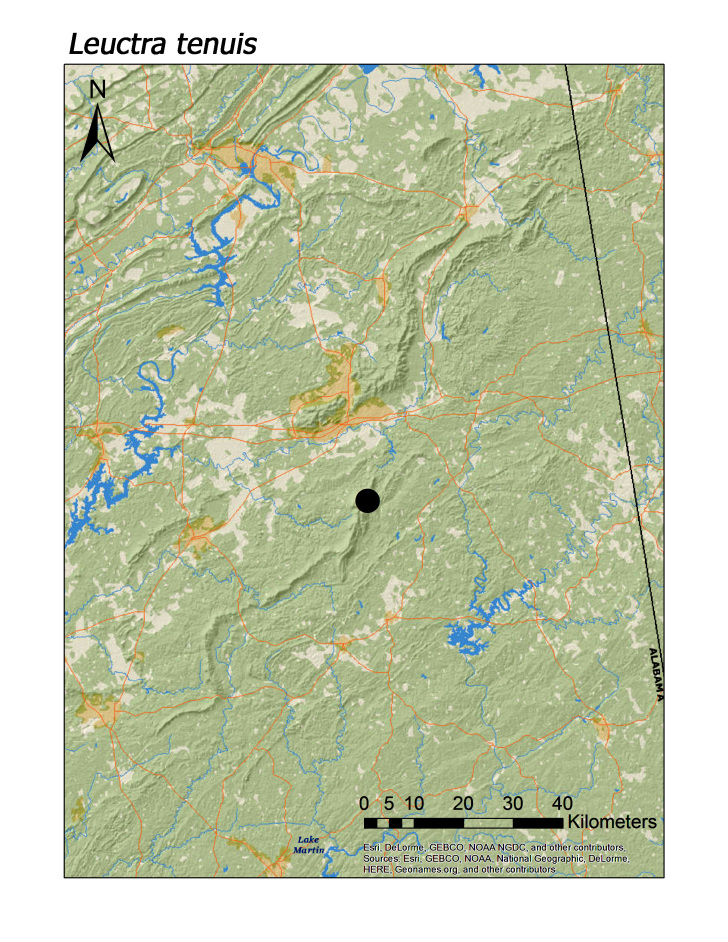
*L. tenuis*

**Figure 9a.**
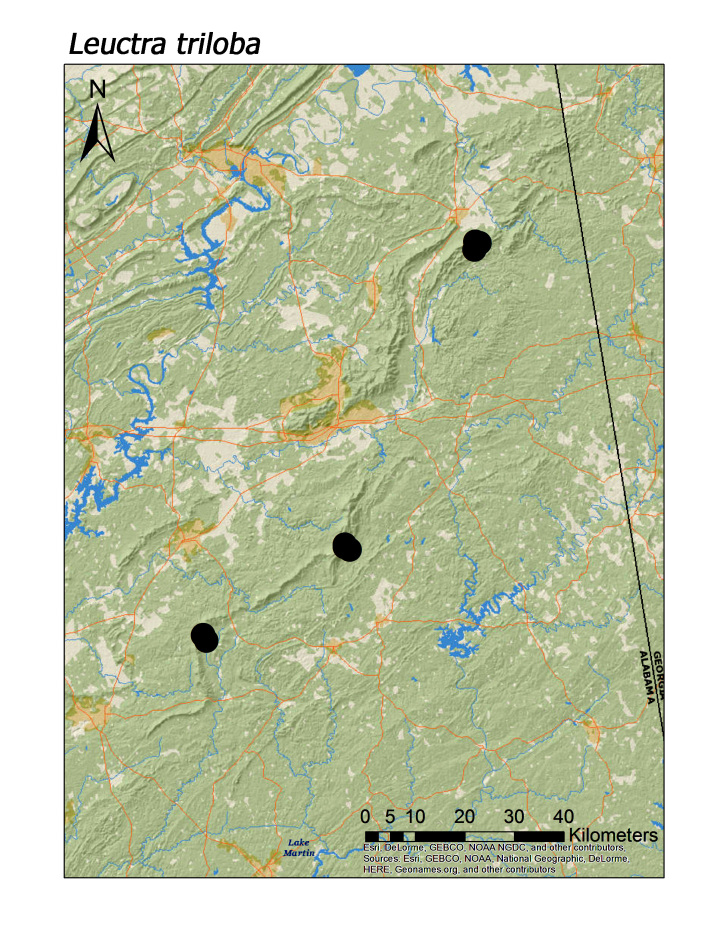
*Leuctra triloba*

**Figure 9b.**
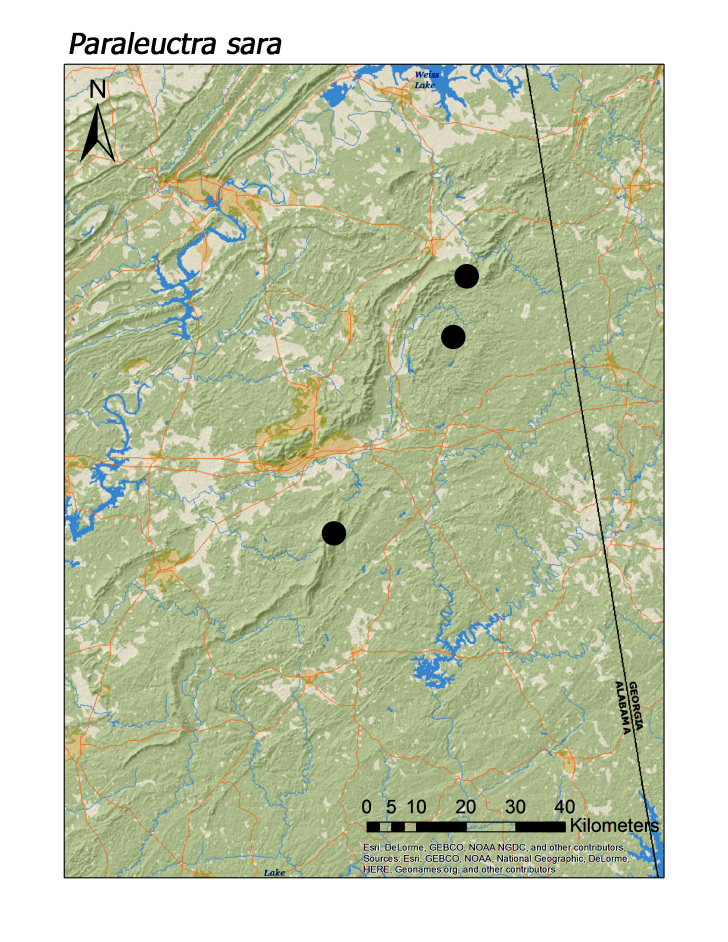
*Paraleuctra sara*

**Figure 9c.**
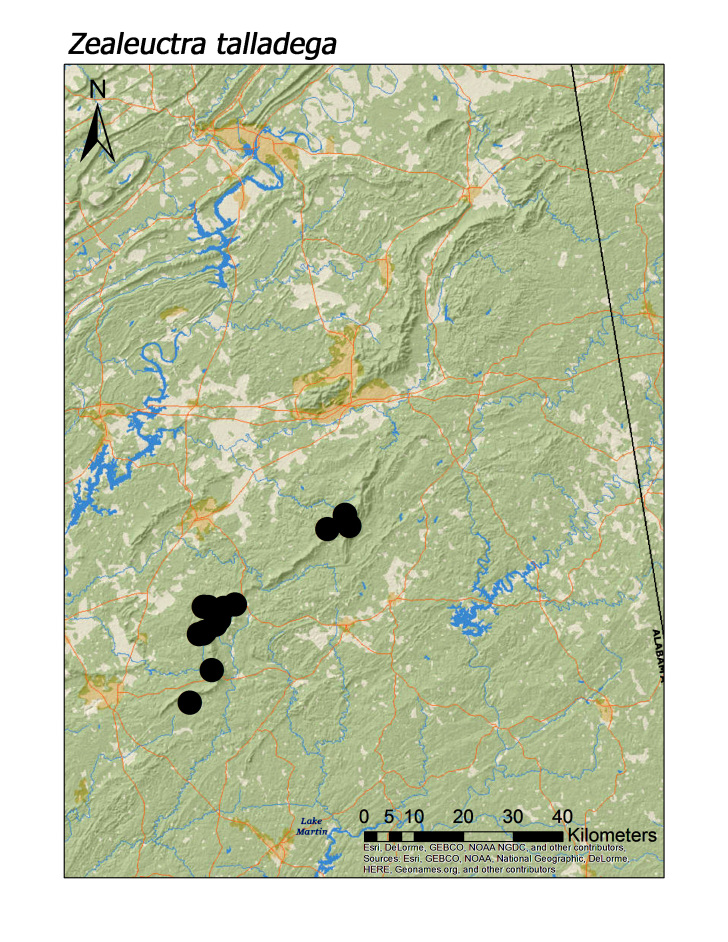
*Zealeuctra talladega*

**Figure 9d.**
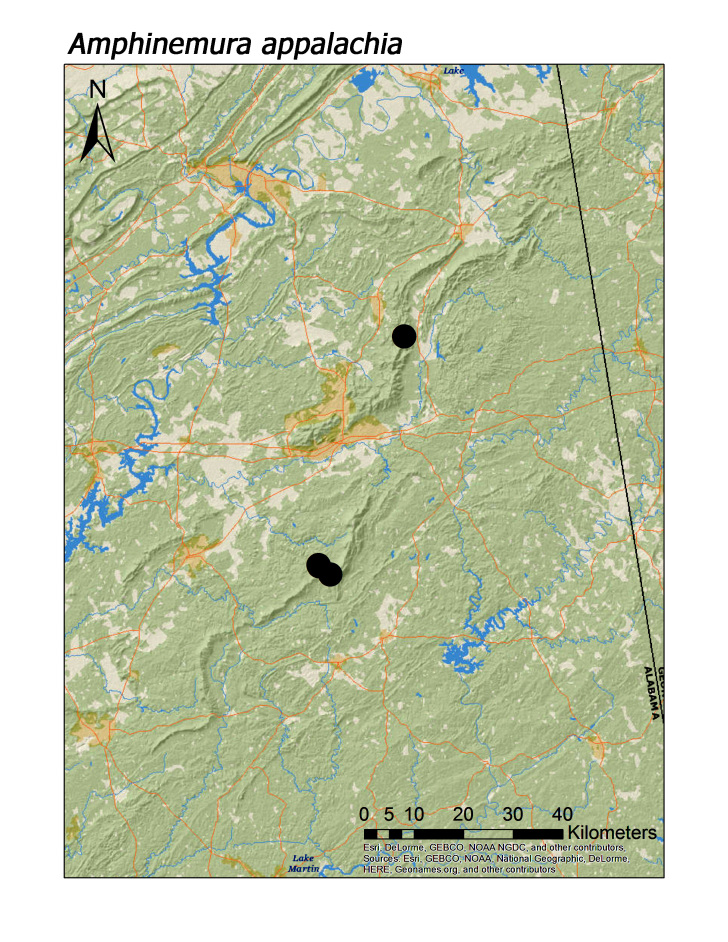
*Amphinemura appalachia*

**Figure 10a.**
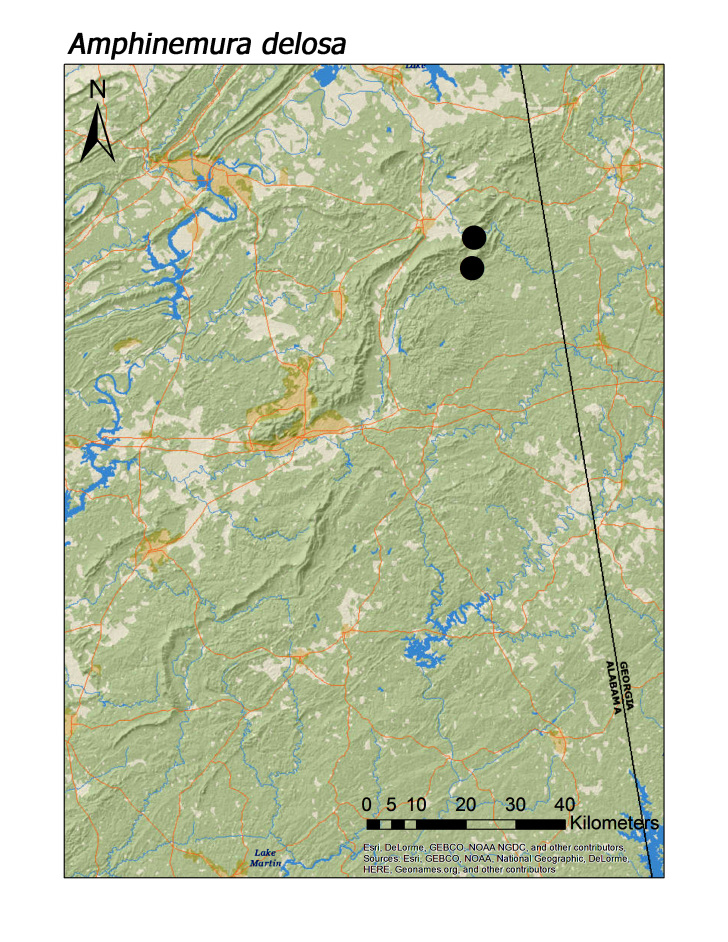
*Amphinemura delosa*

**Figure 10b.**
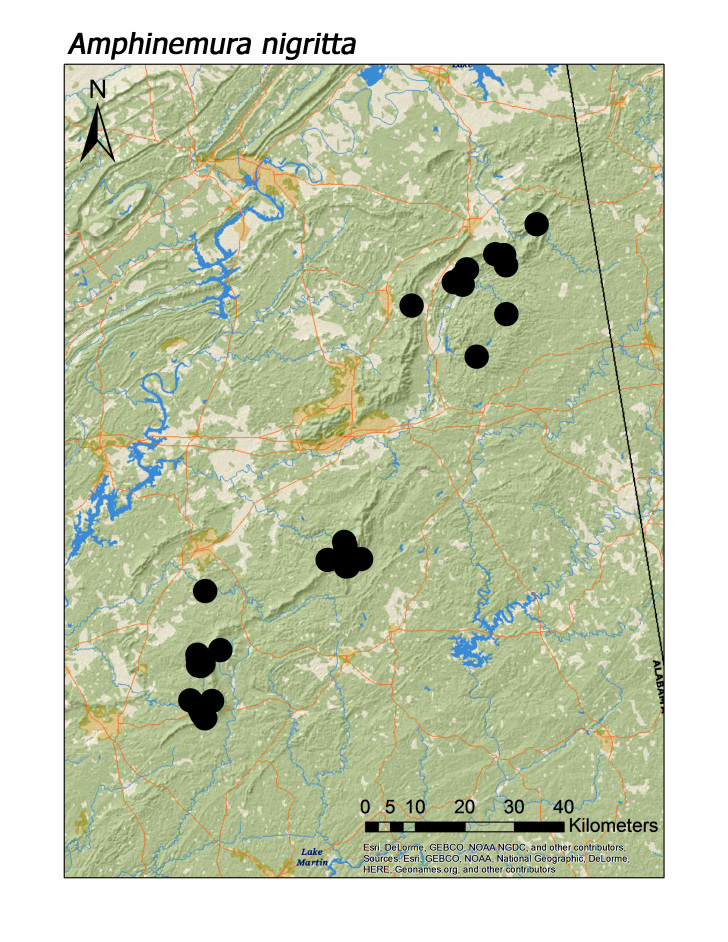
*A. nigritta*

**Figure 10c.**
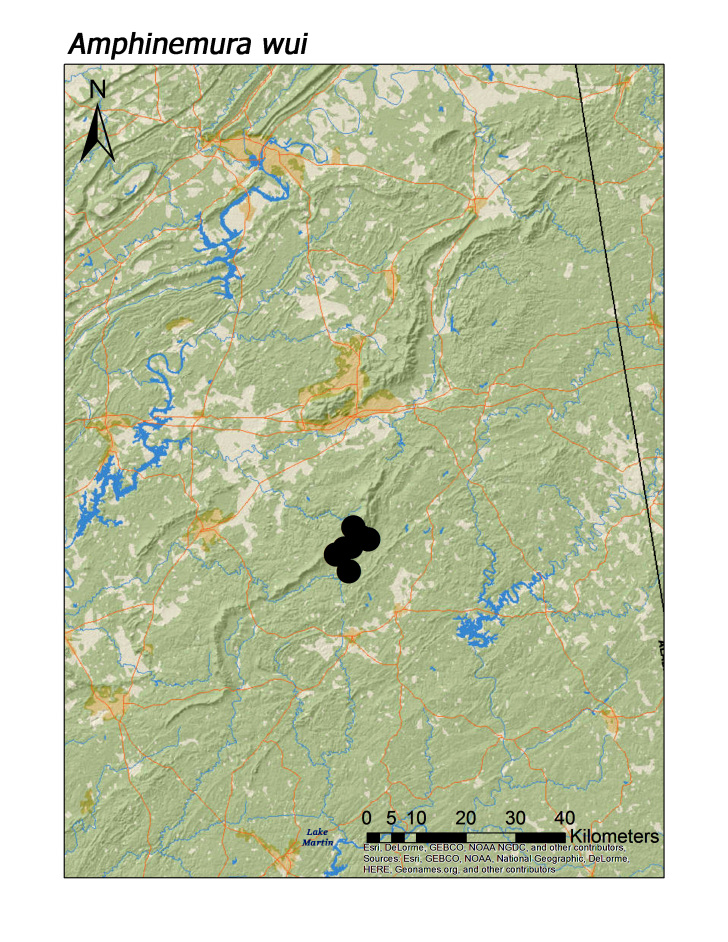
*A. wui*

**Figure 10d.**
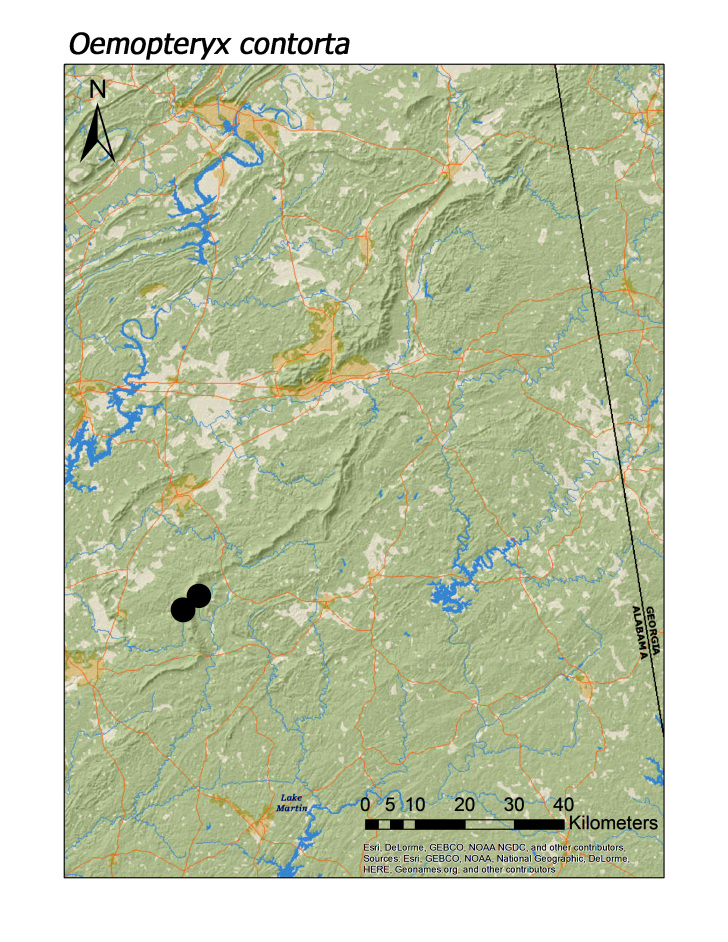
*Oemopteryx contorta*

**Figure 11a.**
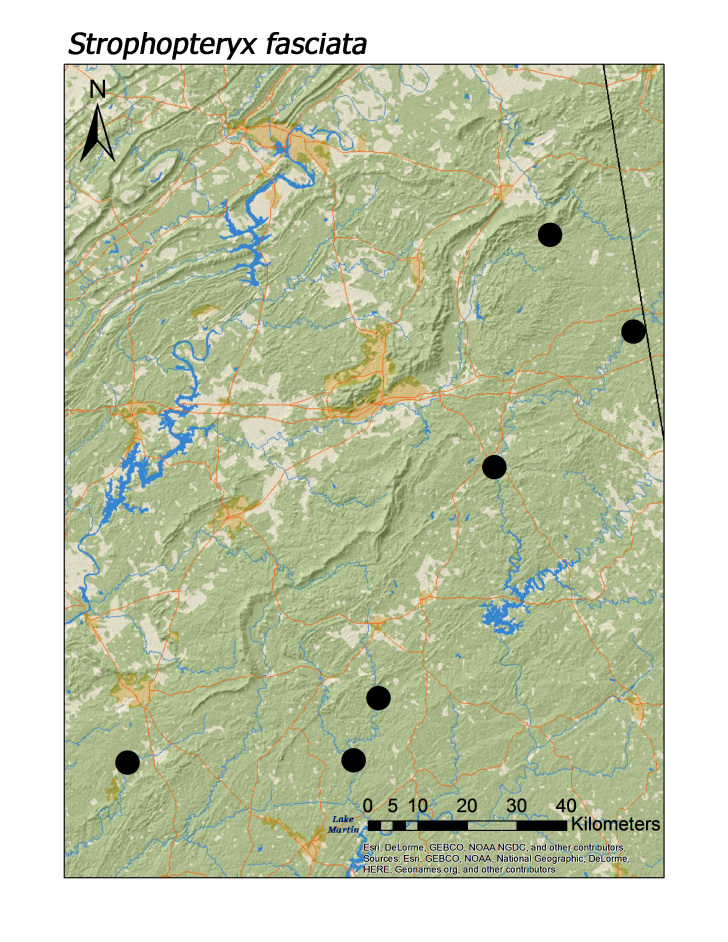
*Strophopteryx fasciata*

**Figure 11b.**
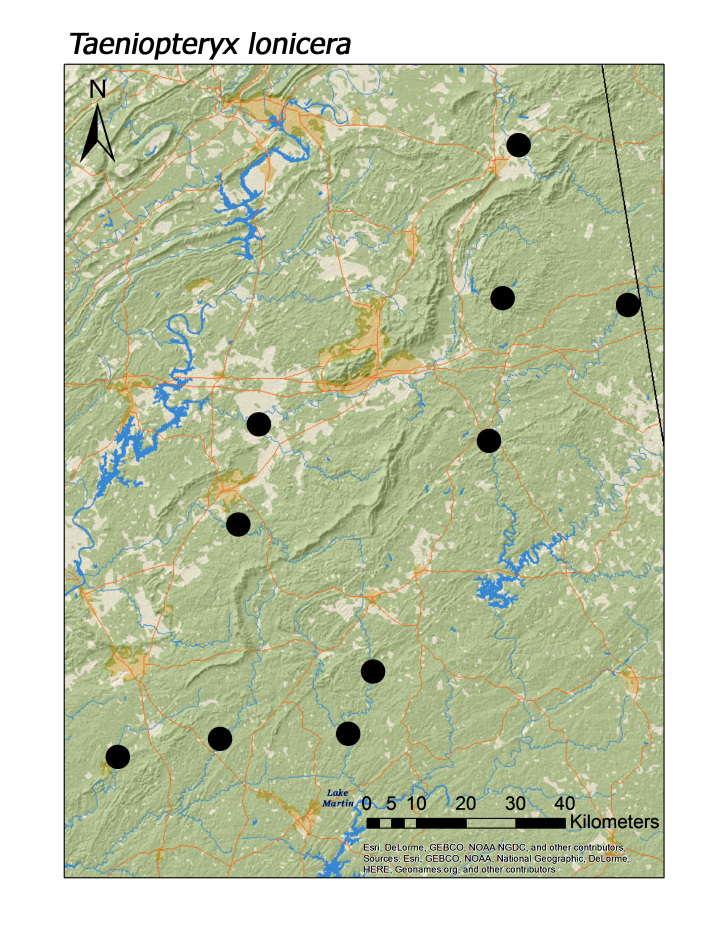
*Taeniopteryx lonicera*

**Figure 11c.**
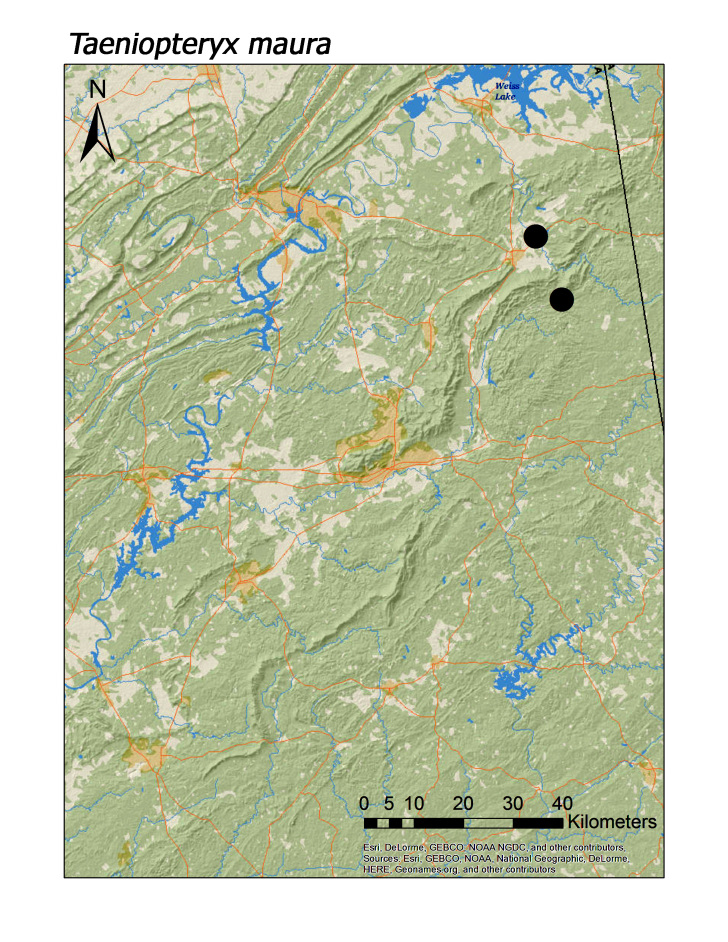
*T. maura*

**Figure 11d.**
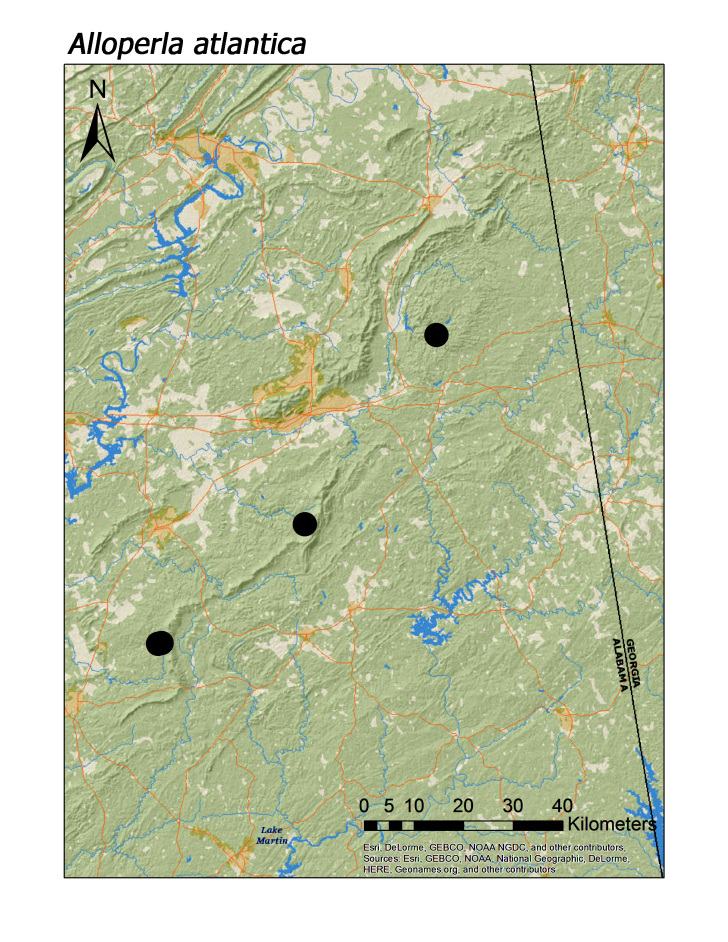
*Alloperla atlantica*

**Figure 12a.**
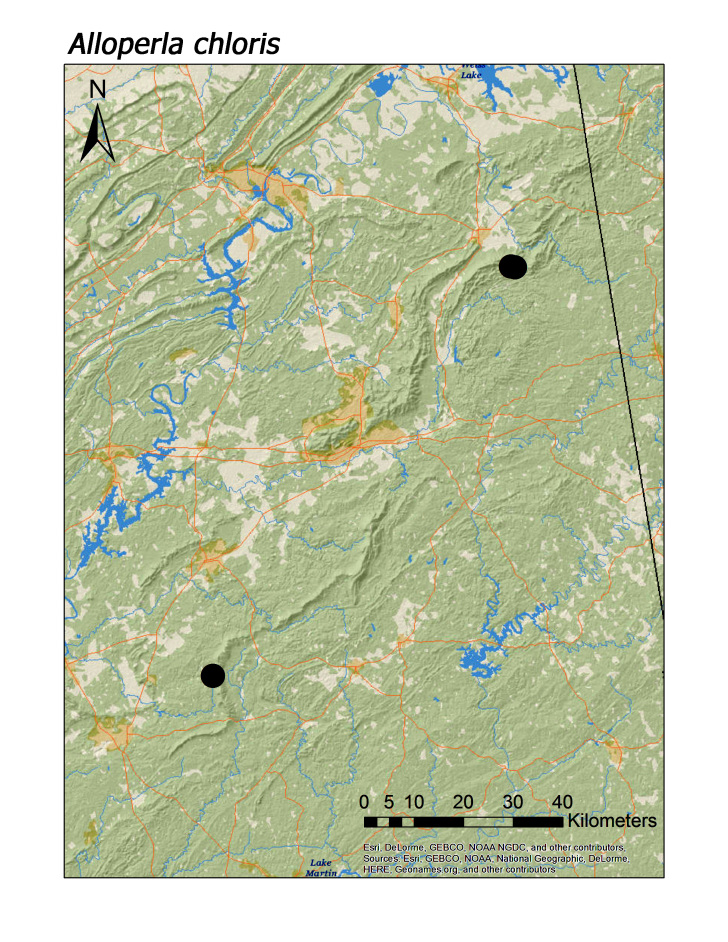
*Alloperla chloris*

**Figure 12b.**
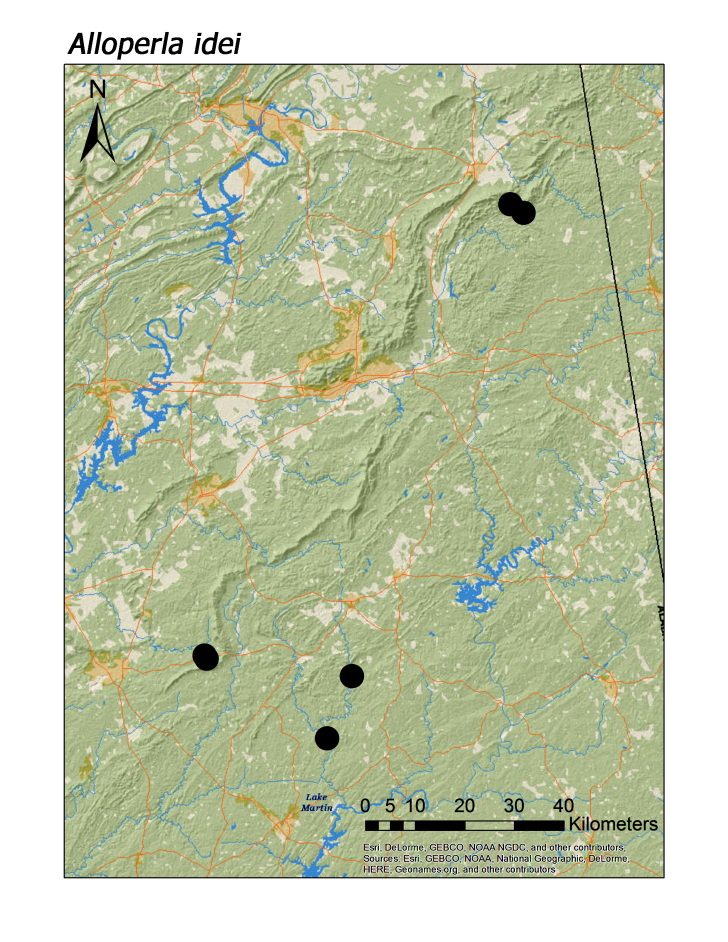
*A. idei*

**Figure 12c.**
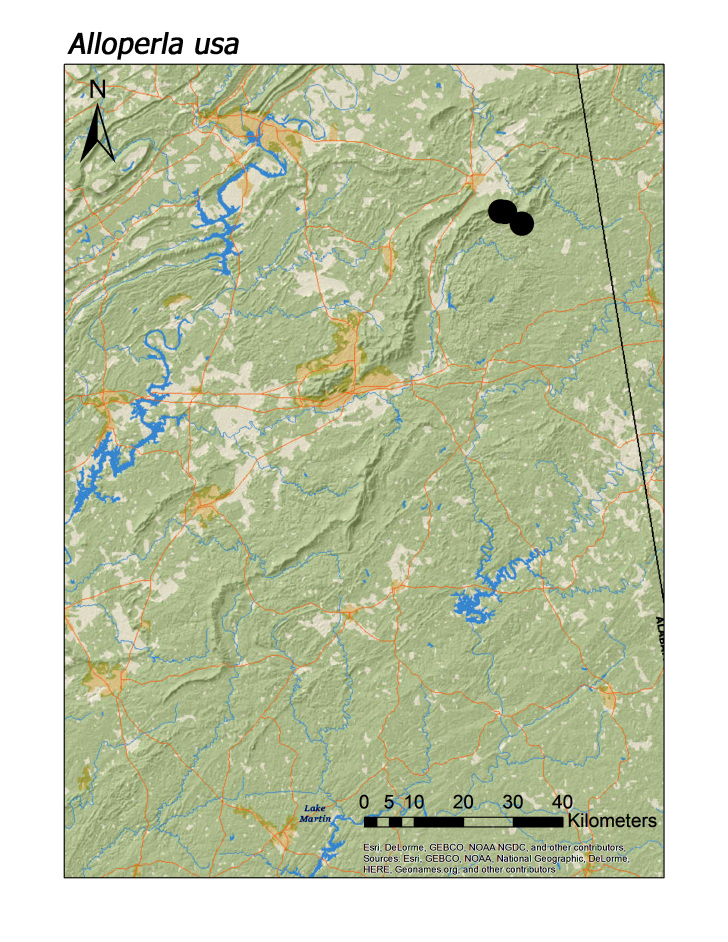
*A. usa*

**Figure 12d.**
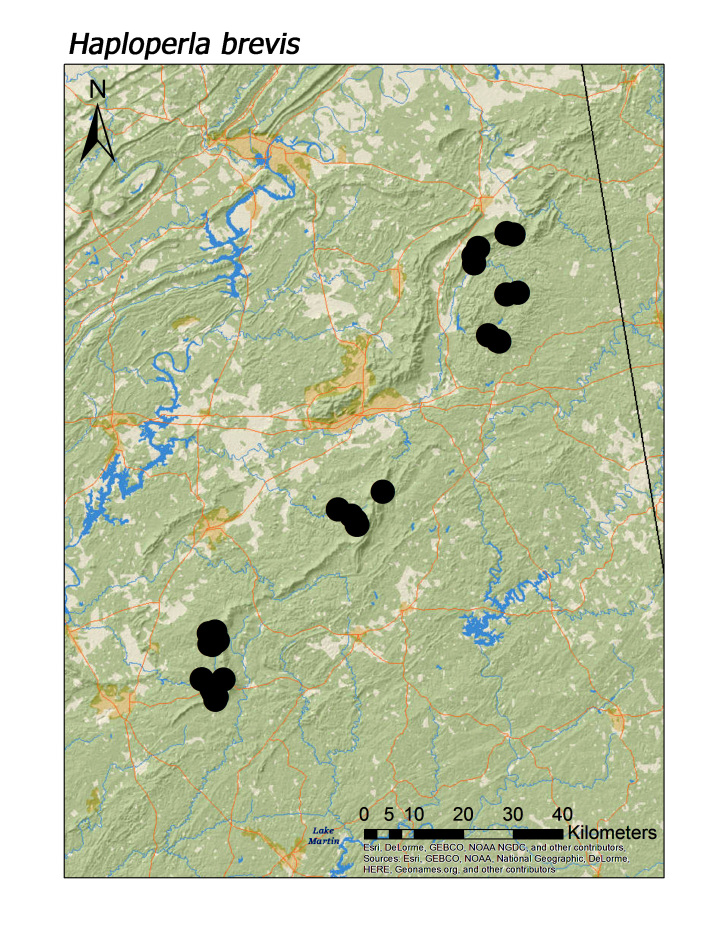
*Haploperla brevis*

**Figure 13a.**
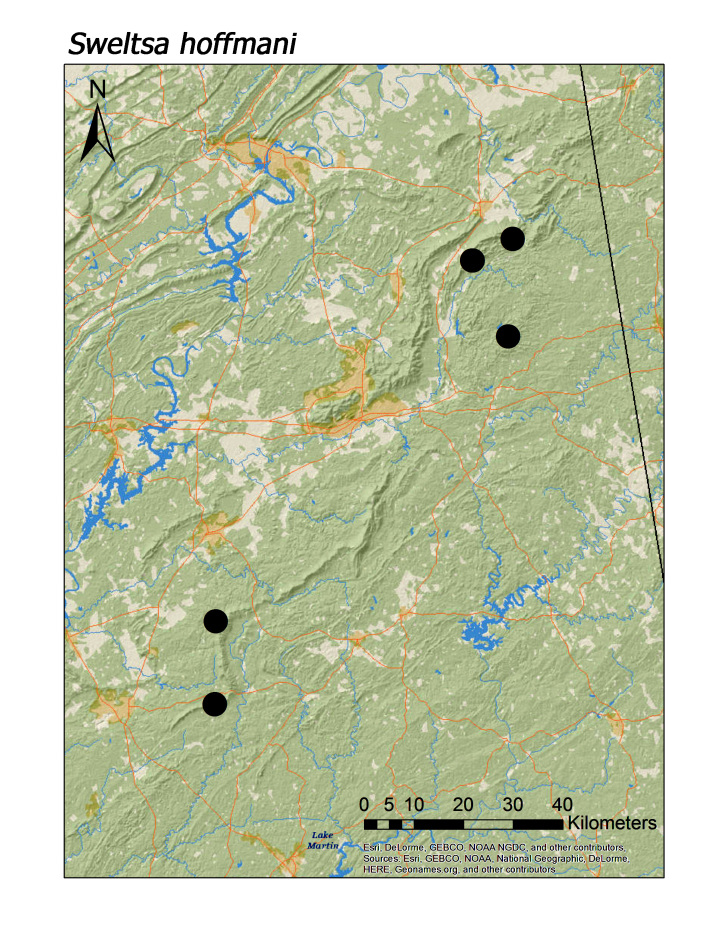
*Sweltsa hoffmani*

**Figure 13b.**
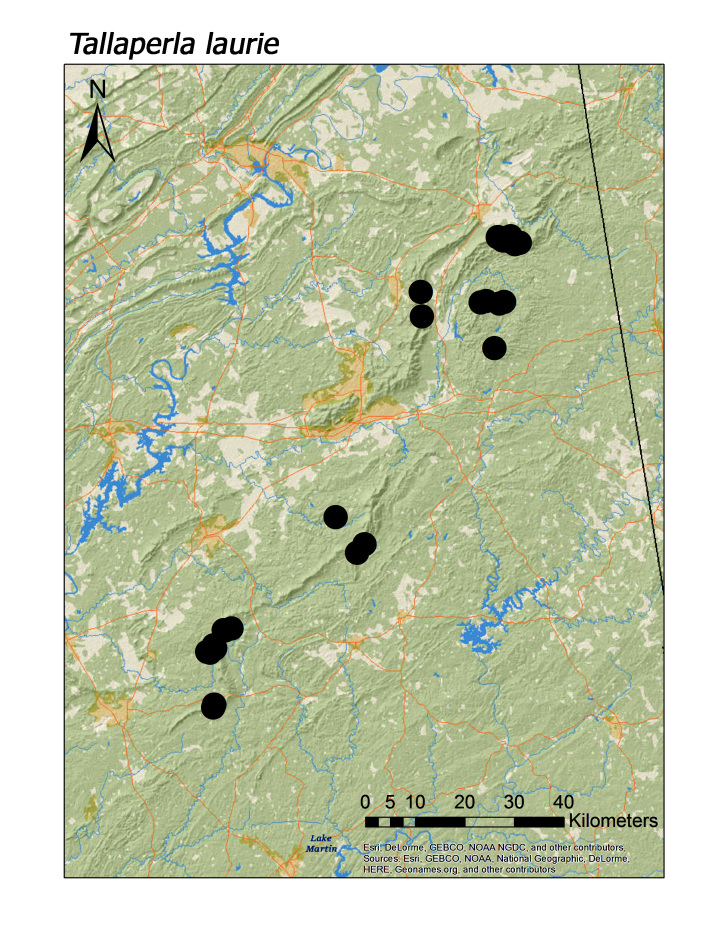
*Tallaperla laurie*

**Figure 13c.**
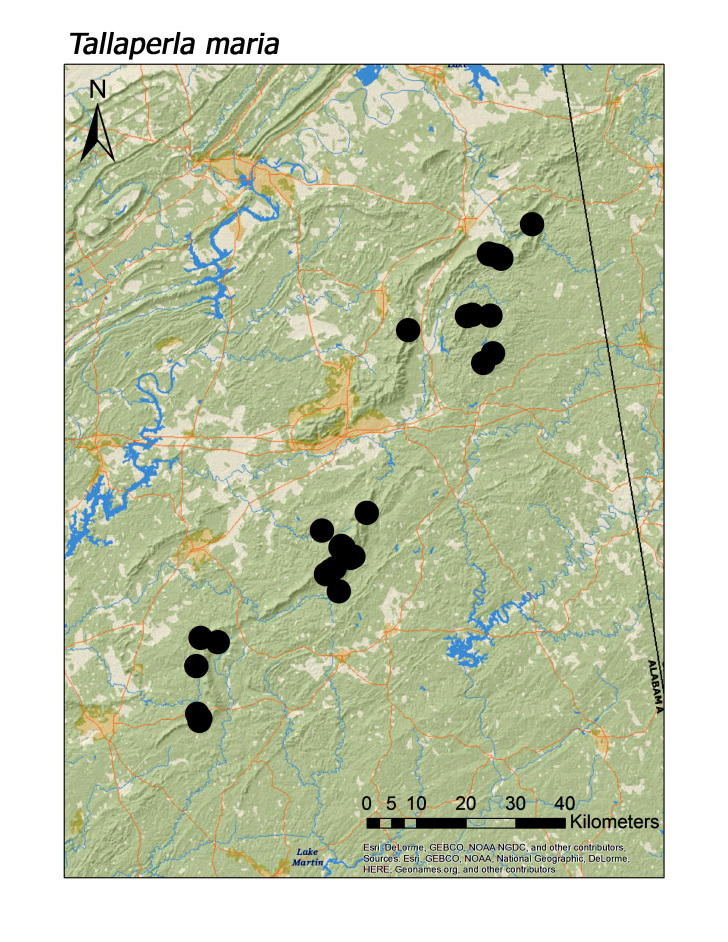
*T. maria*

**Figure 13d.**
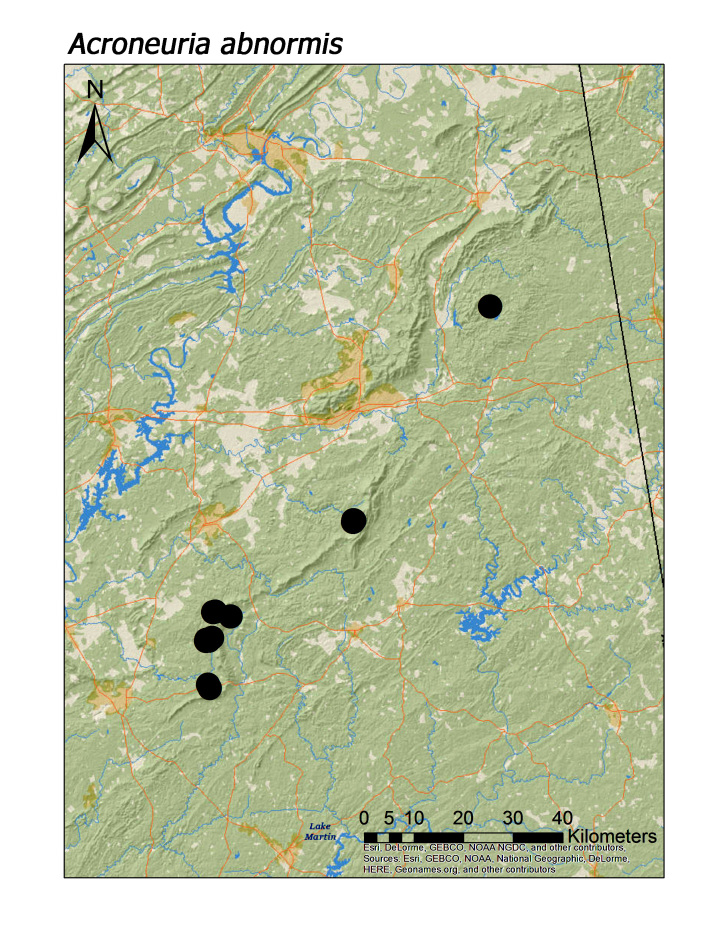
*Acroneuria abnormis*

**Figure 14a.**
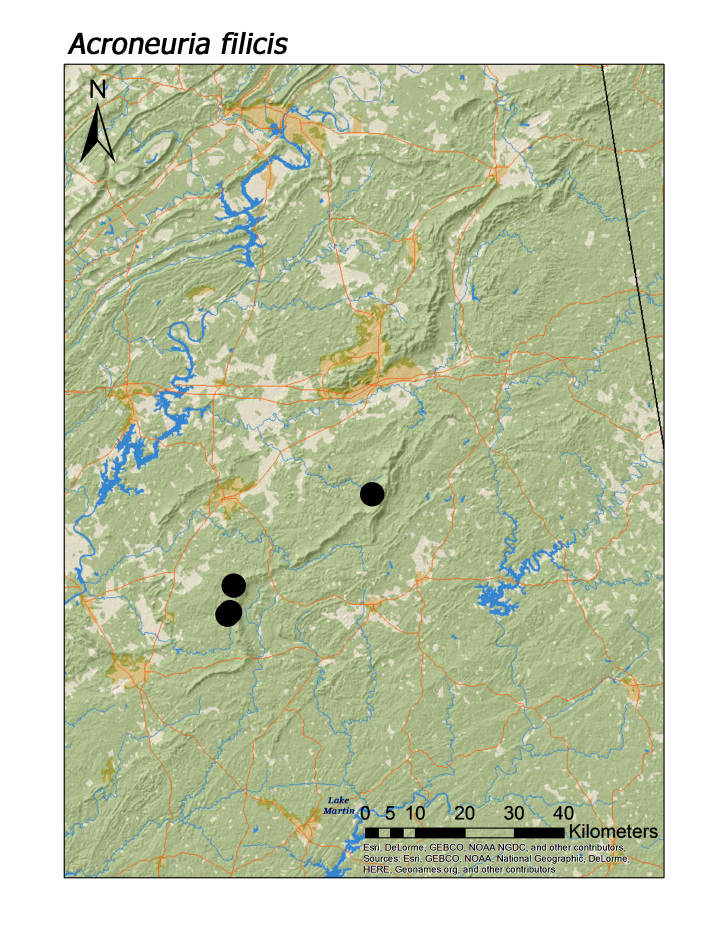
*Acroneuria filicis*

**Figure 14b.**
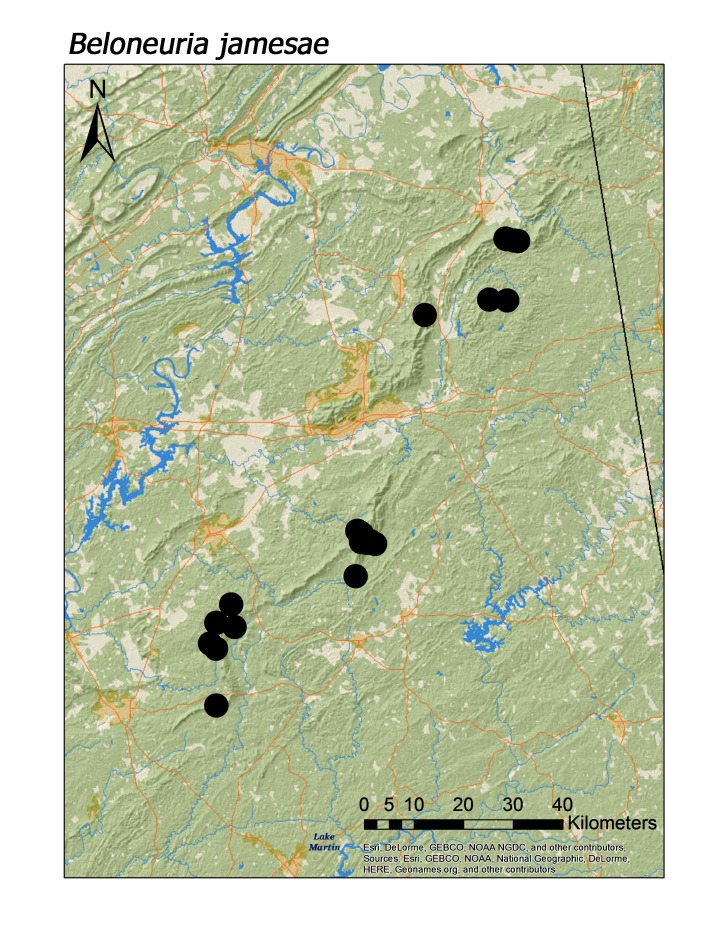
*Beloneuria jamesae*

**Figure 14c.**
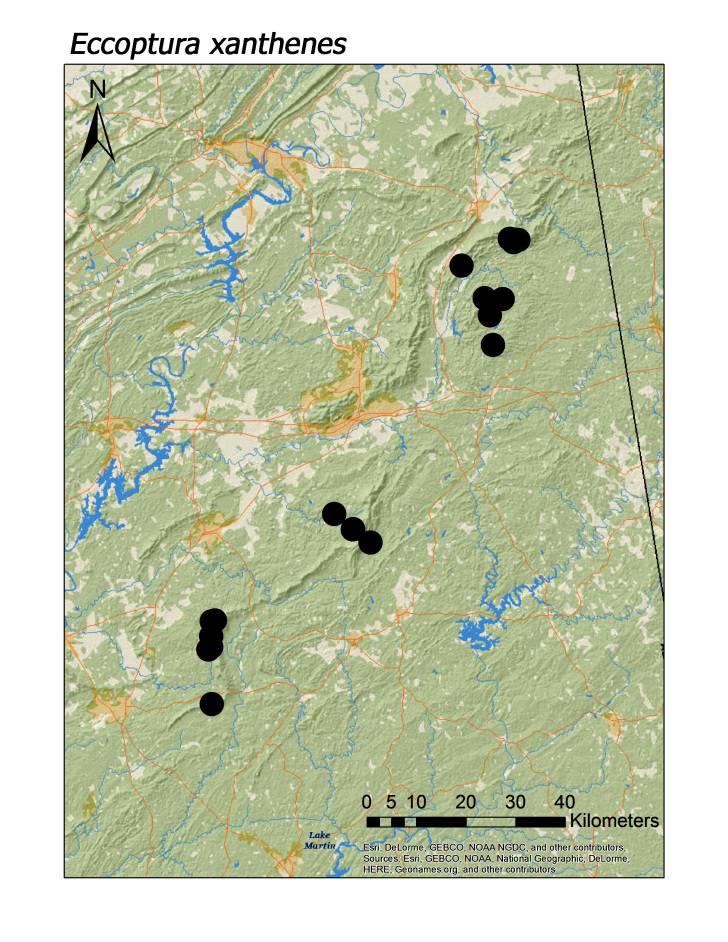
*Eccoptura xanthenes*

**Figure 14d.**
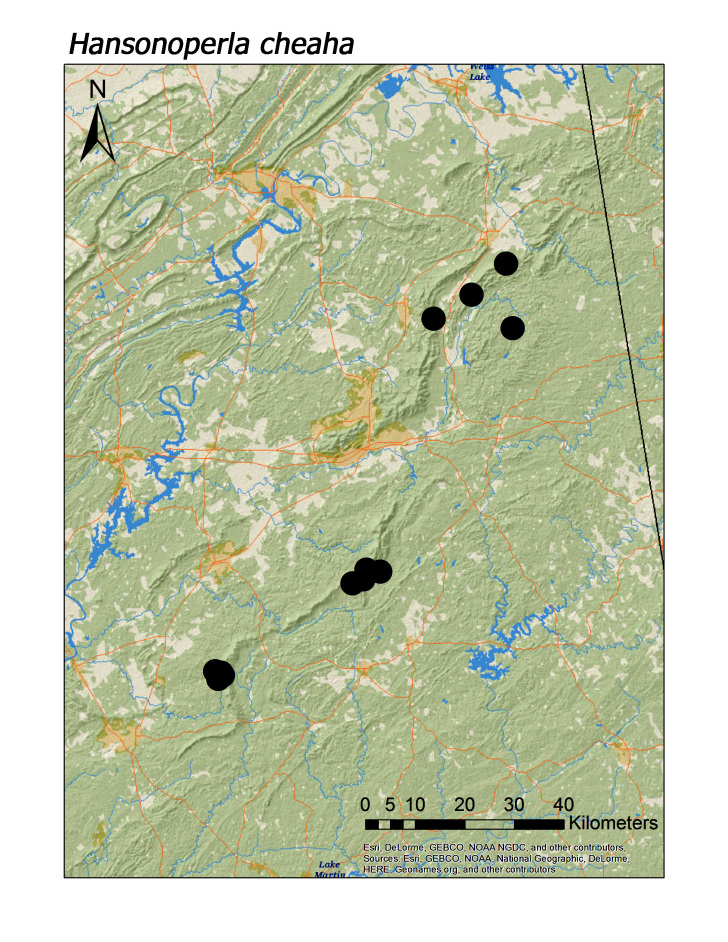
*Hansonoperla cheaha*

**Figure 15a.**
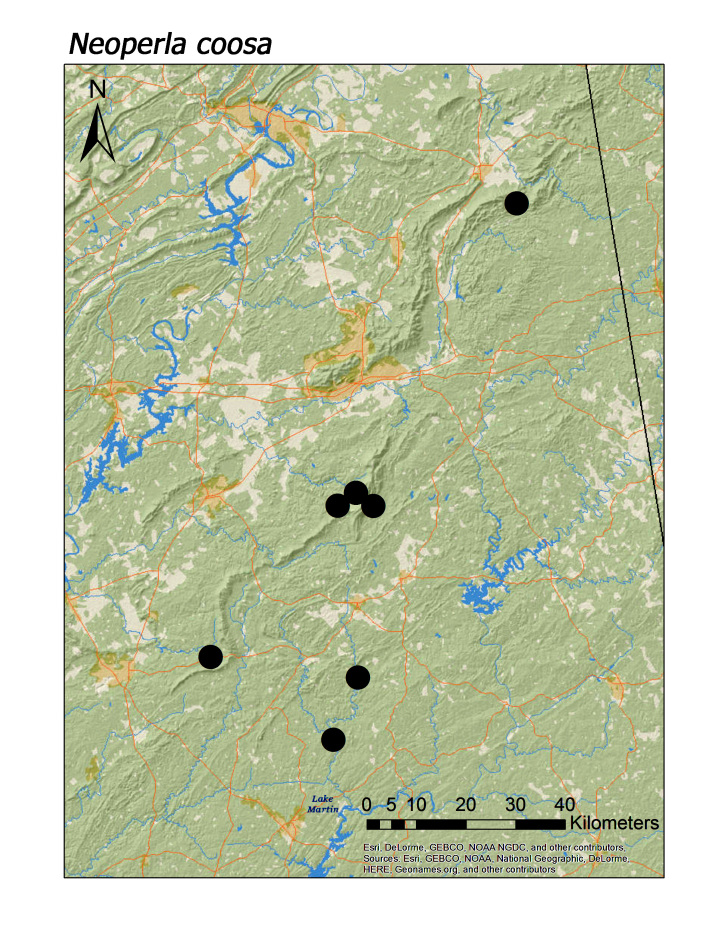
*Neoperla coosa*

**Figure 15b.**
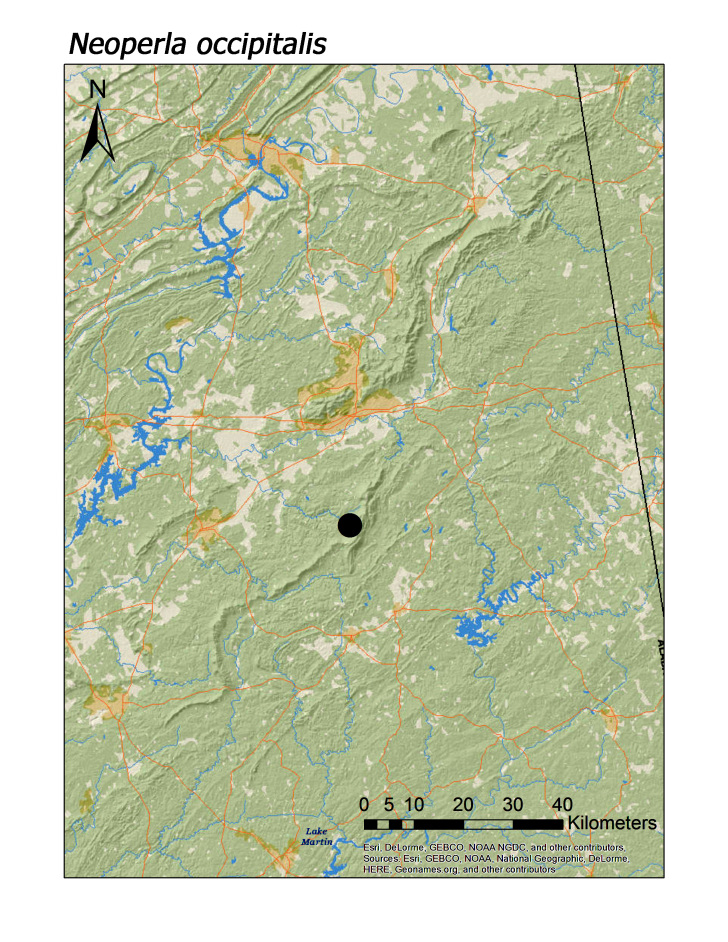
*N. occipitalis*

**Figure 15c.**
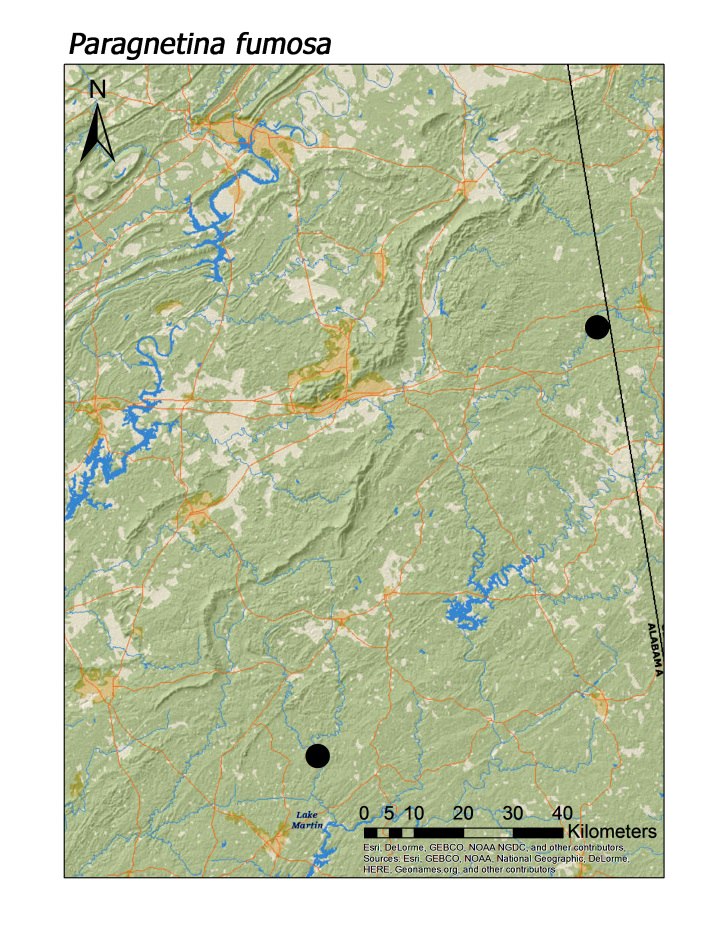
*Paragnetina fumosa*

**Figure 15d.**
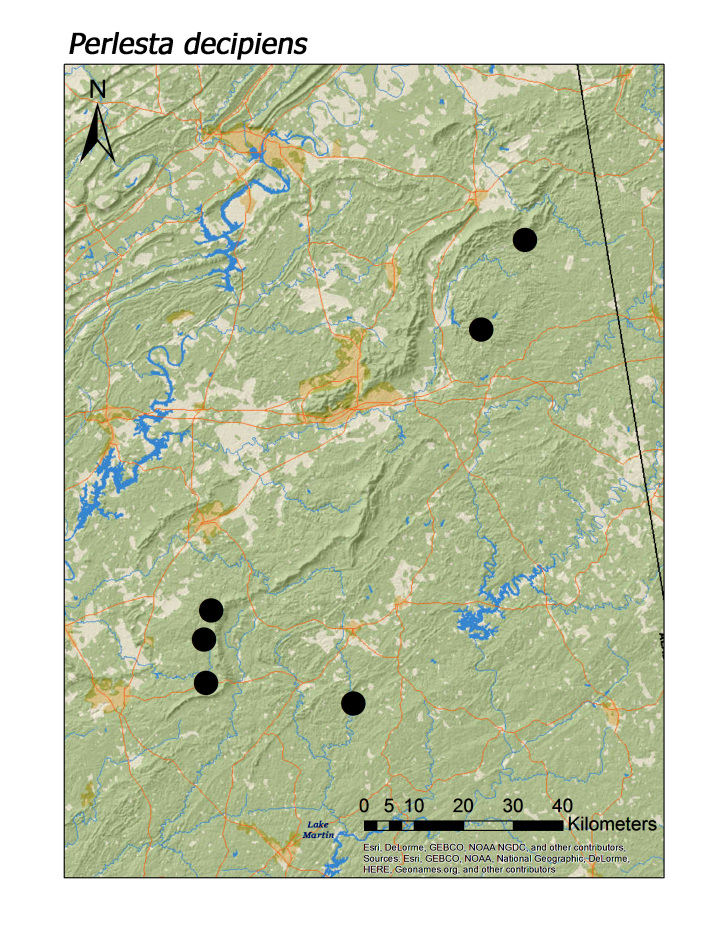
*Perlesta decipiens*

**Figure 16a.**
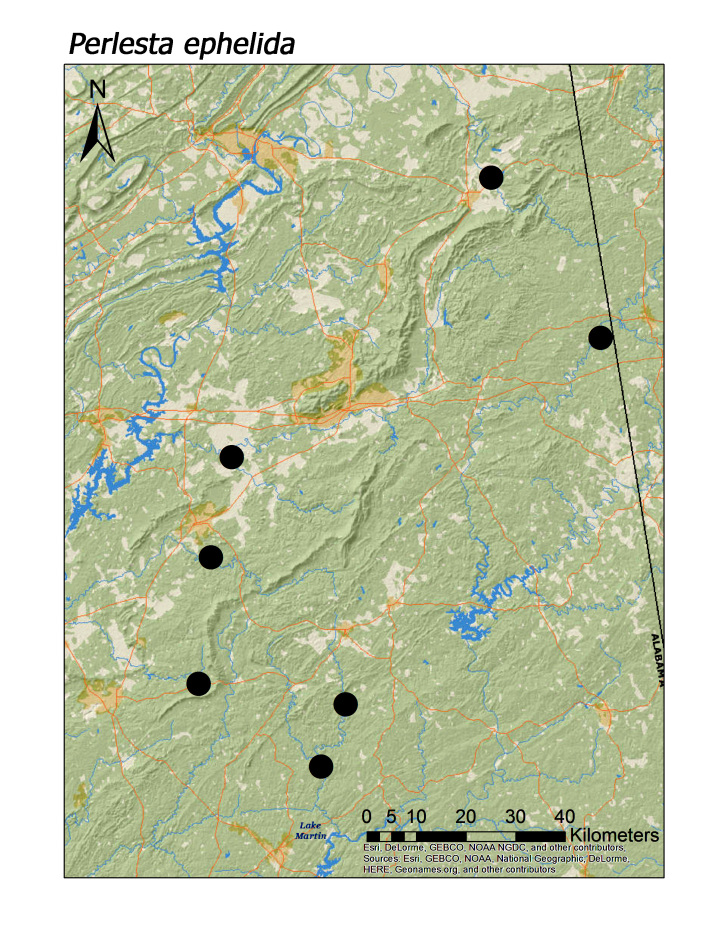
*Perlesta ephelida*

**Figure 16b.**
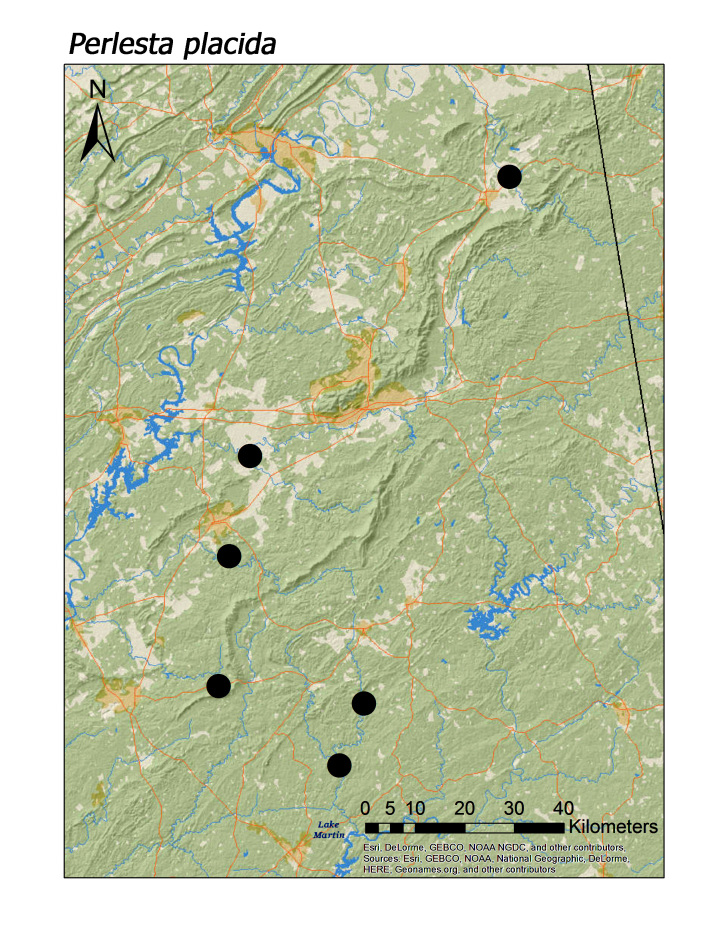
*P. placida*

**Figure 16c.**
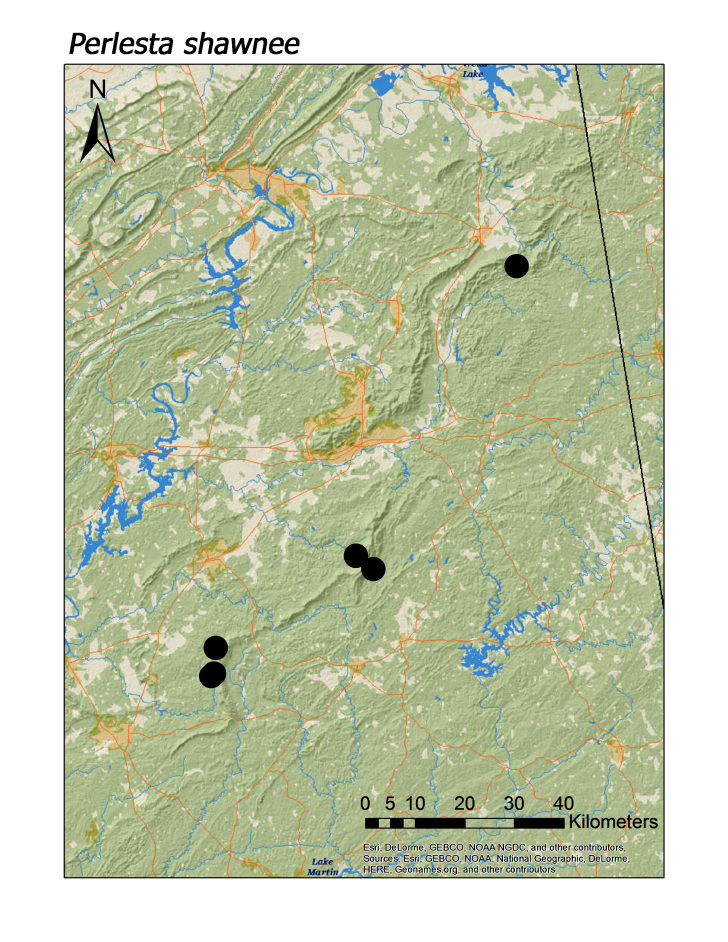
*P. shawnee*

**Figure 16d.**
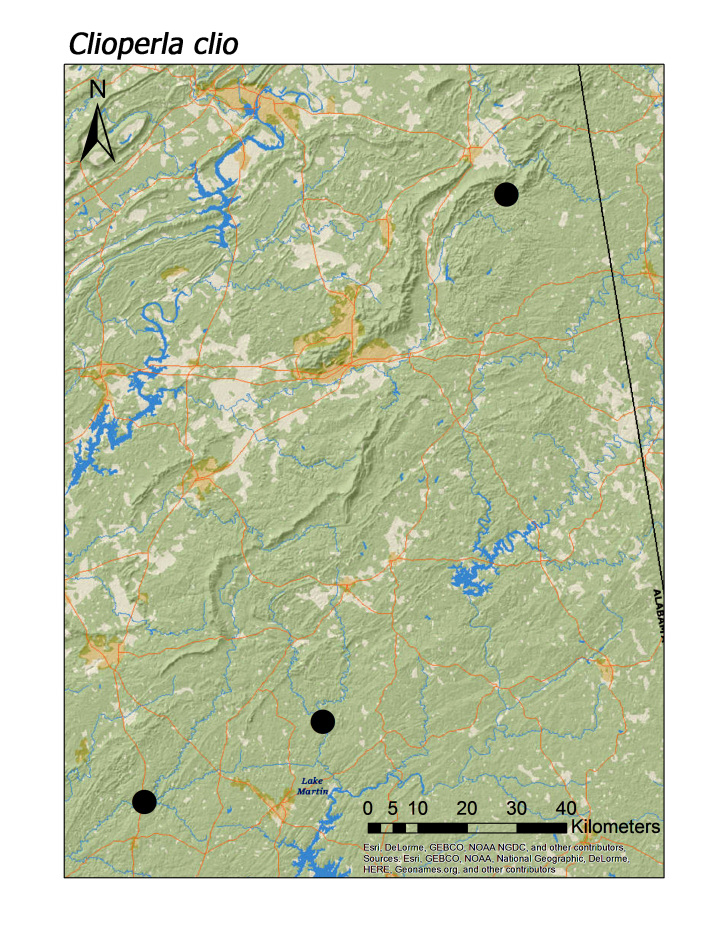
*Clioperla clio*

**Figure 17a.**
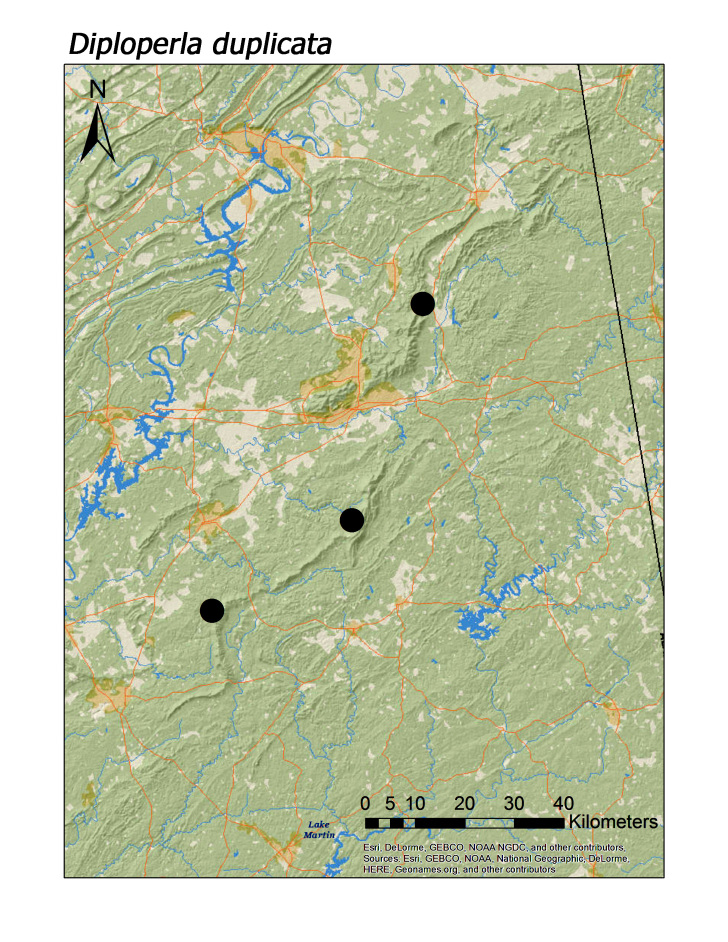
*Diploperla duplicata*

**Figure 17b.**
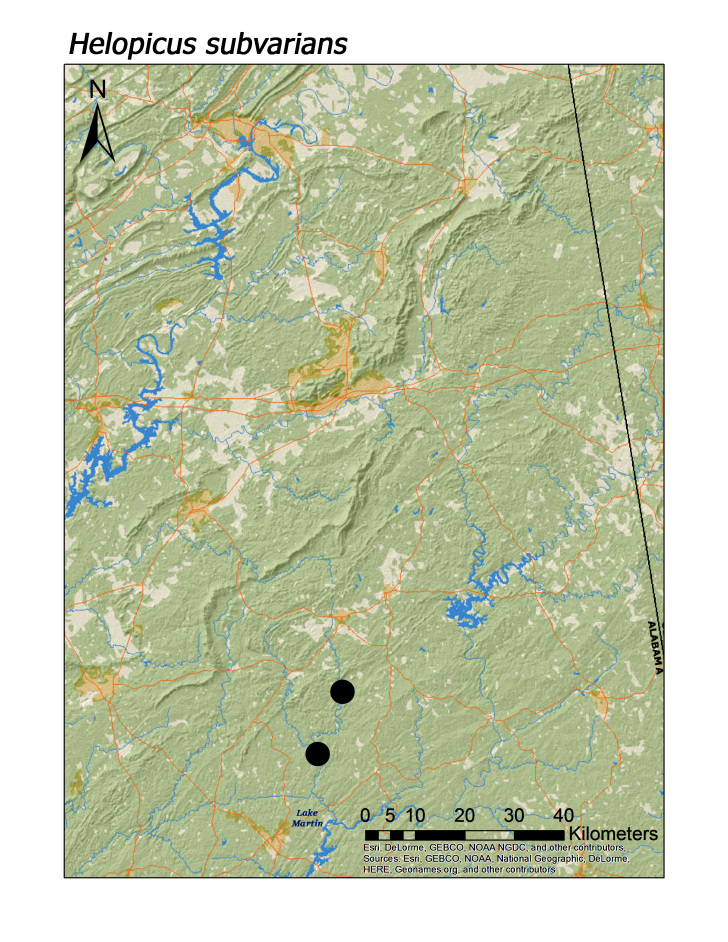
*Helopicus subvarians*

**Figure 17c.**
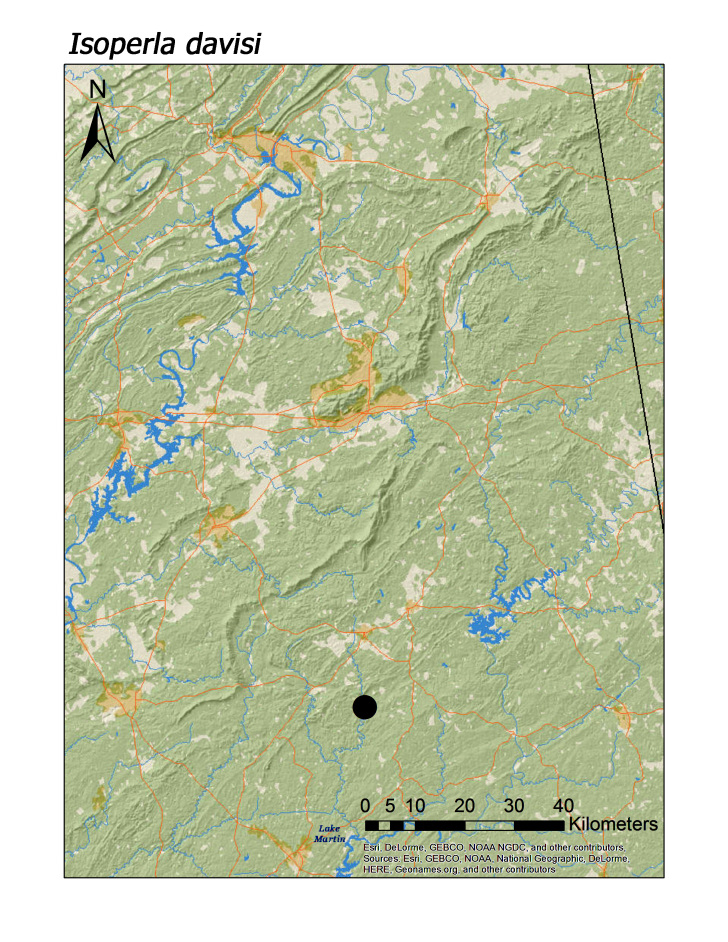
*Isoperla davisi*

**Figure 17d.**
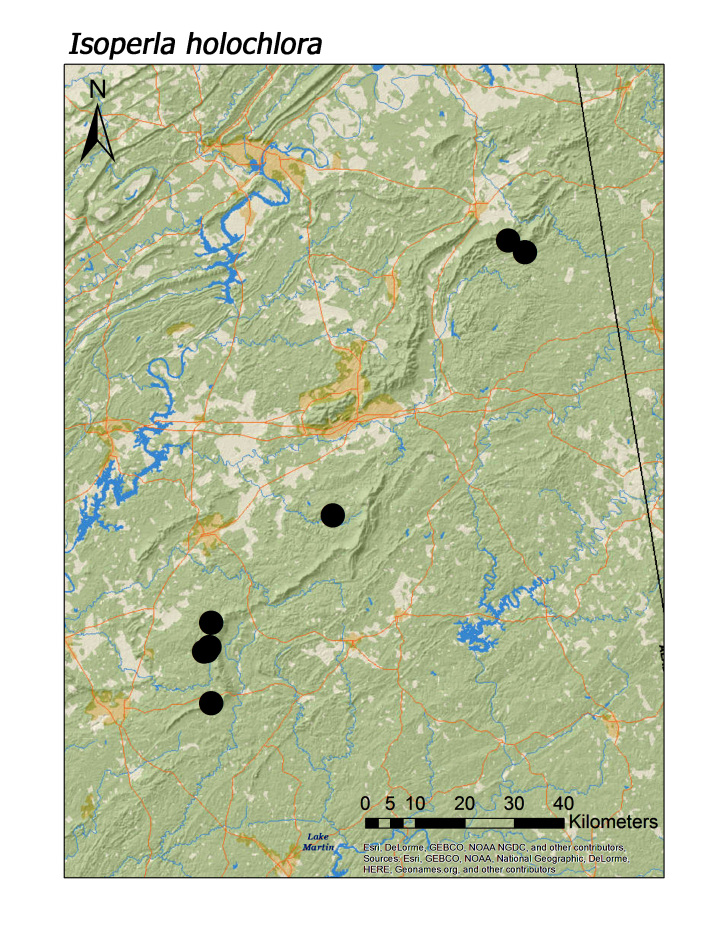
*I. holochlora*

**Figure 18a.**
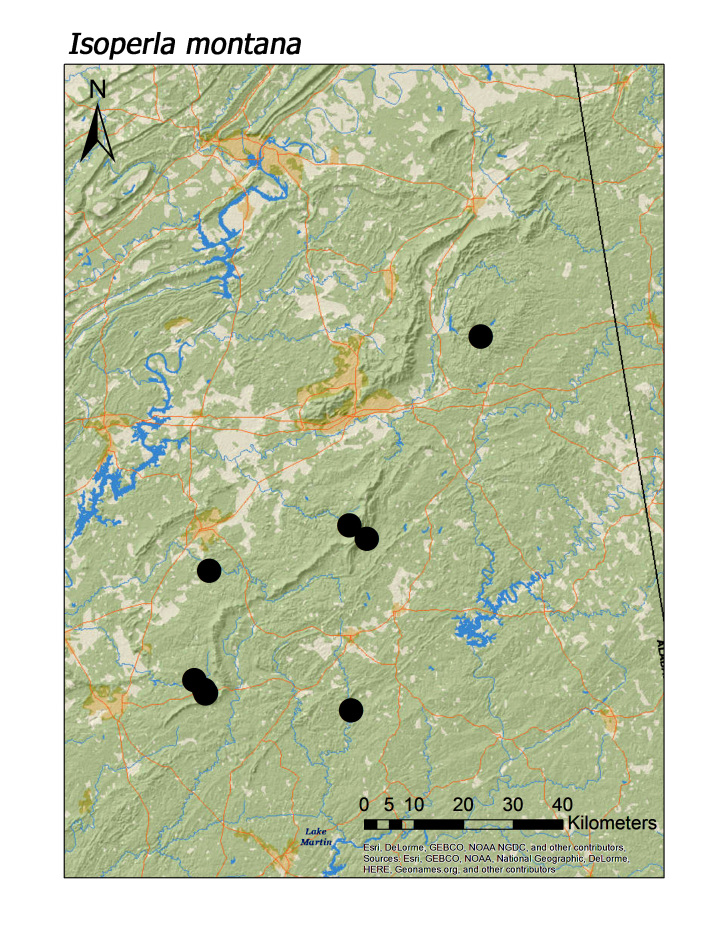
*Isoperla montana*

**Figure 18b.**
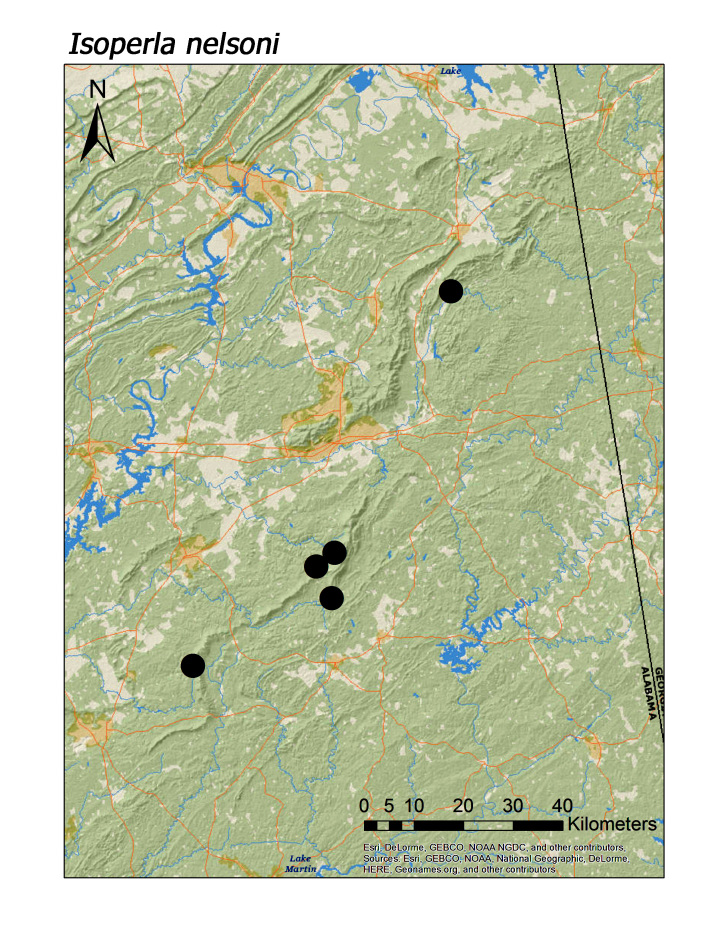
*I. nelsoni*

**Figure 18c.**
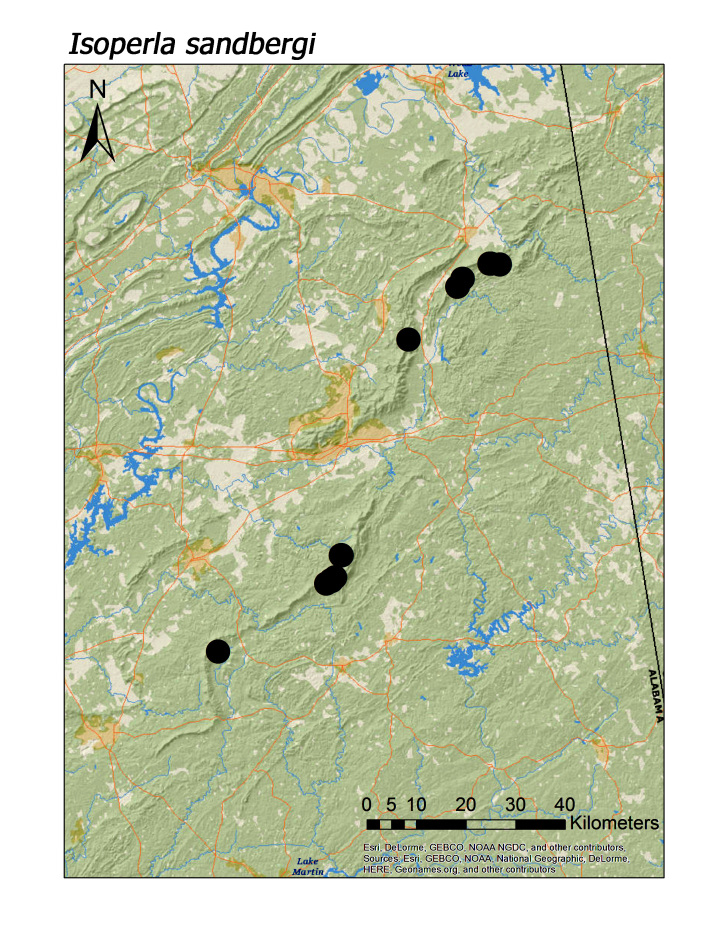
*I. sandbergi*

**Figure 18d.**
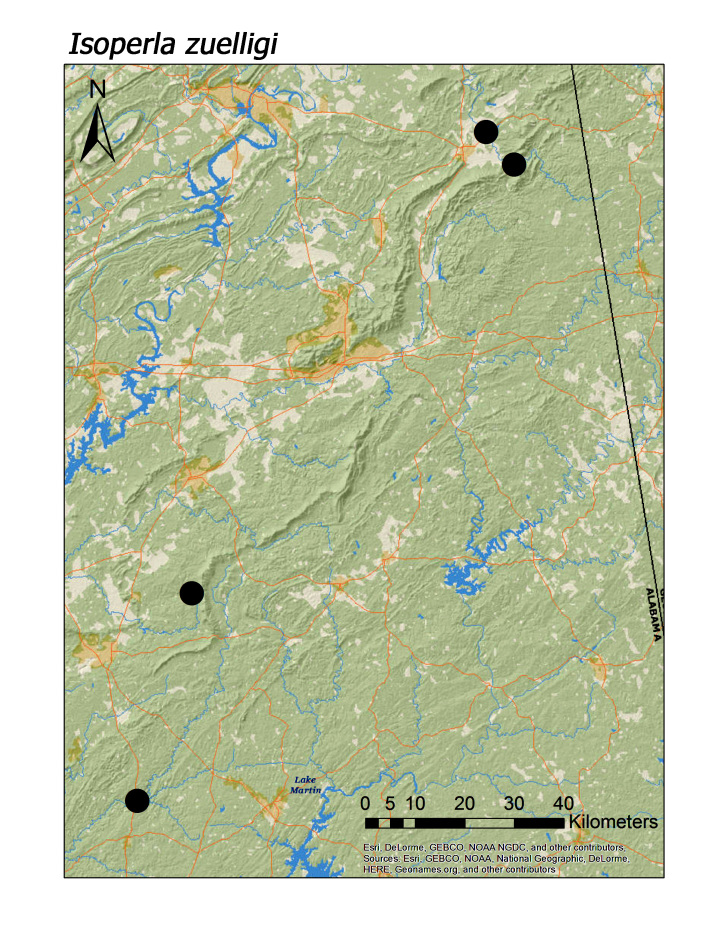
*I. zuelligi*

**Figure 19a.**
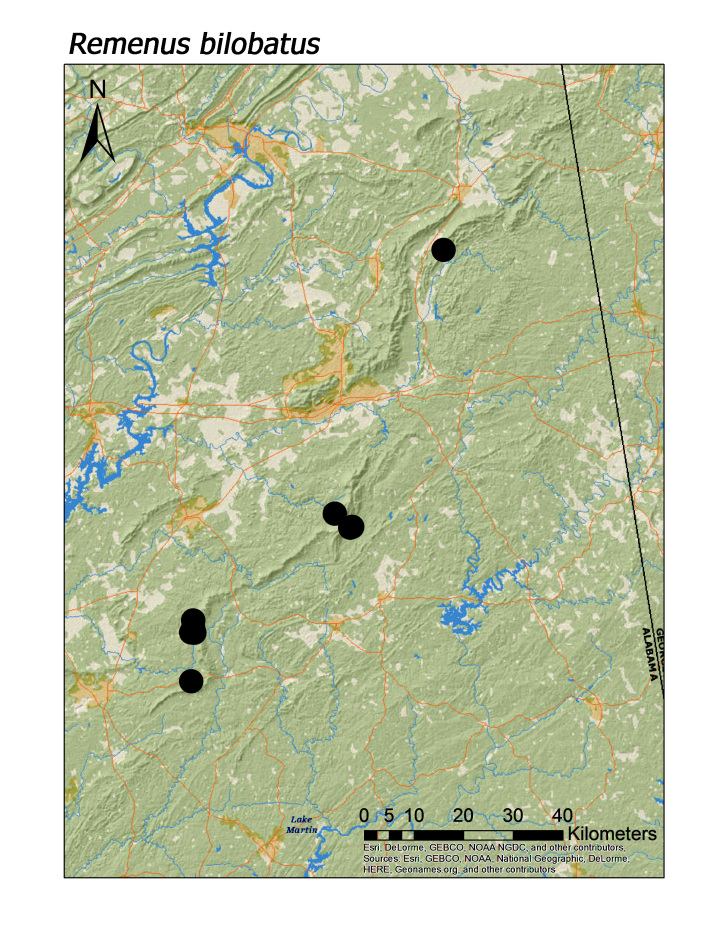
*Remenus bilobatus*

**Figure 19b.**
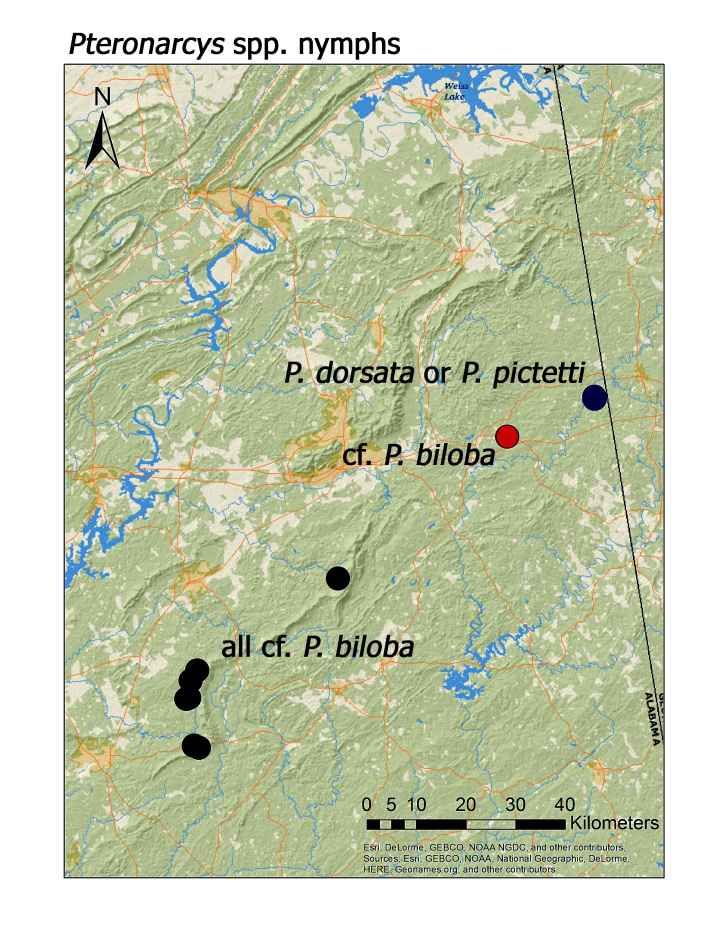
*Pteronarcys nymphs*

**Table 1.** Stonefly species plotted in James (1972) with a Talladega Mountain region distribution.

**Species**	**Comments**	**Collected 2003–2012**
**Family Capniidae**		
*Allocapnia aurora* Ricker, 1952		Yes
*Allocapnia mystica* Frison, 1929		Yes
*Allocapnia recta* (Claassen, 1924)		Yes
*Allocapnia rickeri* Frison, 1942		Yes
*Allocapnia virginiana* Frison, 1942		Yes
**Family Leuctridae**		
*Leuctra alabama* n. sp.	Informal manuscript name; maybe plotted in error	No
*Leuctra alexanderi* Hanson, 1941	Now referable to *Leuctra crossi* James, 1976	No
*Leuctra alta* n. sp.	Informal manuscript name	Yes
*Leuctra biloba* Claassen, 1923		No
*Leuctra cottaquilla* n. sp.	Informal manuscript name	Yes
*Leuctra ferruginea* (Walker, 1852)		Yes
*Leuctra moha* Ricker, 1952		No
*Leuctra tenuis* (Pictet, 1841)		Yes
**Family Nemouridae**		
*Amphinemura delosa* (Ricker, 1952)	Listed as *Nemoura delosa* Ricker	Yes
*Amphinemura nigritta* (Provancher, 1876)	Listed as *Nemoura nigritta* Provancher	Yes
**Family Taeniopterygidae**		
*Strophopteryx fasciata* (Burmeister, 1839)	Listed as *Brachyptera fasciata* (Burmeister)	Yes
*Taeniopteryx lonicera* Ricker and Ross, 1968		Yes
*Taeniopteryx maura* (Pictet, 1841)		Yes
**Family Chloroperlidae**		
*Haploperla brevis* (Banks, 1895)	Listed as *Hastaperla brevis* (Banks)	Yes
*Sweltsa hoffmani* Kondratieff and Kirchner, 2009	Listed as *Alloperla mediana* Banks	Yes
**Family Peltoperlidae**		
*Tallaperla maria* (Needham and Smith, 1916)	Listed as *Peltoperla maria* Needham and Smith	Yes
**Family Perlidae**		
*Acroneuria abnormis* (Newman, 1838)		Yes
*Beloneuria jamesae* Stark and Szczytko, 1976	Listed as *Beloneuria georgiana* (Banks)	Yes
*Eccoptura xanthenes* (Newman, 1838)	Listed as *Acroneuria xanthenes* (Newman)	Yes
*Neoperla clymene* (Newman, 1839)	Referable to one of several *Neoperla* species	No
*Paragnetina fumosa* (Banks, 1902)		Yes
*Perlesta placida* (Hagen, 1861)	Referable to several species, including the true *P. placida*	Yes
*Perlinella drymo* (Newman, 1839)		No
**Family Perlodidae**		
*Isoperla davisi* n.sp.	Informal manuscript name	Yes
*Isoperla holochlora* Klapálek, 1923		Yes
*Remenus bilobatus* (Needham and Claassen, 1925)	Listed as *Isogenus bilobatus* (Needham and Claassen)	Yes
**Family Pteronarcyidae**		
*Pteronarcys biloba* Newman, 1838	Questionable; recorded only from one nymph.	Yes (also as nymphs)

**Table 2.** Stonefly species with type localities in the Talladega Mountain region.

**Species**
**Family Capniidae**
*Allocapnia menawa* Grubbs and Sheldon, 2008
*Allocapnia muskogee* Grubbs and Sheldon, 2008
**Family Leuctridae**
*Leuctra alta* James, 1974
*Leuctra cottaquilla* James, 1974
*Leuctra crossi* James, 1976
*Leuctra pinhoti* Grubbs and Sheldon, 2009
*Zealeuctra talladega* Grubbs, 2005
**Family Perlidae**
*Beloneuria jamesae* Stark and Szczytko, 1976
*Hansonoperla cheaha* Kondratieff and Kirchner, 1996
**Family Perlodidae**
*Isoperla davisi* James, 1974
*Isoperla sandbergi* Szczytko and Kondratieff, 2015

**Table 3.** Trips to the Talladega Mountain region made independently by SAG and ALS in 2003–2012.

**Year**	**Month**	**Collector**
2003	February	SAG
2004	May	SAG
2005	March	SAG
2005	May	ALS
2005	October	ALS
2006	January	ALS
2006	May	ALS
2006	June	ALS
2006	December	ALS
2007	March	ALS
2007	June	SAG
2007	December	SAG
2007	December	ALS
2008	January	SAG
2008	April	ALS
2008	May	SAG
2008	July	SAG
2009	February	SAG
2010	April	SAG
2011	November	ALS
2012	March	SAG
2012	April	ALS
2012	April	SAG
2012	May	ALS
2012	July	ALS
2012	September	ALS

**Table 4.** List and summary information of stonefly species collected by SAG and ALS from the Talladega Mountain region in 2003–2012. The "**" refers to four species previously described as new during this study period 2003-2012. **Bold type** indicates species that may be endemic.

**Species**	**Total no. collections**	**Total no. localities**
**Family Capniidae**		
*Allocapnia aurora* Ricker, 1952	1	1
***Allocapnia menawa*** Grubbs and Sheldon, 2008**	3	2
*Allocapnia muskogee* Grubbs and Sheldon, 2008**	4	4
*Allocapnia mystica* Frison, 1929	1	1
*Allocapnia recta* (Claassen, 1924)	32	25
*Allocapnia rickeri* Frison, 1942	7	6
*Allocapnia smithi* Ross and Ricker, 1971	13	13
*Allocapnia virginiana* Frison, 1942	8	6
*Nemocapnia carolina* Banks, 1938	4	2
**Family Leuctridae**		
*Leuctra alta* James, 1974	20	19
*Leuctra cottaquilla* James, 1974	8	7
***Leuctra crossi*** James, 1976	66	61
*Leuctra ferruginea* (Walker, 1852)	26	26
*Leuctra grandis* Banks, 1906	31	28
***Leuctra pinhoti*** Grubbs and Sheldon, 2009**	11	11
*Leuctra tenuis* (Pictet, 1841)	1	1
*Leuctra triloba* Claassen, 1923	9	9
*Paraleuctra sara* (Claassen, 1937)	4	4
***Zealeuctra talladega*** Grubbs, 2005**	22	21
**Family Nemouridae**		
*Amphinemura appalachia* Baumann, 1996	5	4
*Amphinemura delosa* (Ricker, 1952)	2	2
*Amphinemura nigritta* (Provancher, 1876)	44	43
*Amphinemura wui* (Claassen, 1936)	9	8
**Family Taeniopterygidae**		
*Oemopteryx contorta* (Needham and Claassen, 1925)	3	3
*Strophopteryx fasciata* (Burmeister, 1839)	8	6
*Taeniopteryx lonicera* Ricker and Ross, 1968	15	10
*Taeniopteryx maura* (Pictet, 1841)	5	2
**Family Chloroperlidae**		
*Alloperla atlantica* Baumann, 1974	4	4
*Alloperla chloris* Frison, 1934	4	4
*Alloperla idei* (Ricker, 1935)	8	7
*Alloperla usa* Ricker, 1952	3	3
*Haploperla brevis* (Banks, 1895)	38	37
*Sweltsa hoffmani* Kondratieff and Kirchner, 2009	8	8
**Family Peltoperlidae**		
*Tallaperla laurie* (Ricker, 1952)	38	38
*Tallaperla maria* (Needham and Smith, 1916)	31	31
**Family Perlidae**		
*Acroneuria abnormis* (Newman, 1838)	18	17
*Acroneuria filicis* Frison, 1942	4	4
***Beloneuria jamesae*** Stark and Szczytko, 1976	26	26
*Eccoptura xanthenes* (Newman, 1838)	22	22
***Hansonoperla cheaha*** Kondratieff and Kirchner, 1996	12	12
*Neoperla coosa* Smith and Stark, 1998	8	7
*Neoperla occipitalis* (Pictet, 1841)	1	1
*Paragnetina fumosa* (Banks, 1902)	3	2
*Perlesta decipiens* (Walsh, 1862)	6	6
*Perlesta ephelida* Grubbs and DeWalt, 2012	8	8
*Perlesta placida* (Hagen, 1861)	6	6
*Perlesta shawnee* Grubbs, 2005	8	7
**Family Perlodidae**		
*Clioperla clio* (Newman, 1839)	3	3
*Diploperla duplicata* (Banks, 1920)	3	3
*Helopicus subvarians* (Banks, 1920)	2	2
*Isoperla davisi* James, 1974	2	1
*Isoperla holochlora* Klapálek, 1923	8	8
*Isoperla montana* (Banks, 1898)	11	11
*Isoperla nelsoni* Szczytko and Kondratieff, 2015	5	5
***Isoperla sandbergi*** Szczytko and Kondratieff, 2015	22	19
*Isoperla zuelligi* Szczytko and Kondratieff, 2015	5	4
*Remenus bilobatus* (Needham and Claassen, 1925)	13	13
**Total number of specimen records**	**696**	
**Total number of specimens**	**3238**	

**Table 5.** Presence of stonefly species as adults from the Talladega Mountain region in 2003–2012. Months were divided into 10 day units. Black shading indicates when adults were collected; gray shading refers to periods when adults were likely present but not collected by the authors.

**Species**	**Month**																																			
	Sep			Oct			Nov			Dec			Jan			Feb			Mar			Apr			May			Jun			Jul			Aug		
*Leuctra cottaquilla*																																				
*Leuctra ferruginea*																																				
*Leuctra tenuis*																																				
*Leuctra triloba*																																				
*Allocapnia muskogee*																																				
*Allocapnia menawa*																																				
*Allocapnia recta*																																				
*Allocapnia aurora*																																				
*Allocapnia virginiana*																																				
*Taeniopteryx maura*																																				
*Allocapnia smithi*																																				
*Taeniopteryx lonicera*																																				
*Zealeuctra talladega*																																				
*Allocapnia mystica*																																				
*Oemopteryx contorta*																																				
*Allocapnia rickeri*																																				
*Strophopteryx fasciata*																																				
*Nemocapnia carolina*																																				
*Clioperla clio*																																				
*Paraleuctra sara*																																				
*Amphinemura appalachia*																																				
*Leuctra alta*																																				
*Leuctra grandis*																																				
*Amphinemura nigritta*																																				
*Isoperla sandbergi*																																				
*Leuctra crossi*																																				
*Isoperla davisi*																																				
*Isoperla montana*																																				
*Isoperla zuelligi*																																				
*Isoperla nelsoni*																																				
*Leuctra pinhoti*																																				
*Alloperla idei*																																				
*Haploperla brevis*																																				
*Sweltsa hoffmani*																																				
*Tallaperla maria*																																				
*Isoperla holochlora*																																				
*Perlesta ephelida*																																				
*Tallaperla laurie*																																				
*Amphinemura delosa*																																				
*Alloperla atlantica*																																				
*Amphinemura wui*																																				
*Perlesta decipiens*																																				
*Remenus bilobatus*																																				
*Hansonoperla cheaha*																																				
*Eccoptura xanthenes*																																				
*Perlesta placida*																																				
*Neoperla occipitalis*																																				
*Alloperla usa*																																				
*Diploperla duplicata*																																				
*Paragnetina fumosa*																																				
*Alloperla chloris*																																				
*Acroneuria abnormis*																																				
*Beloneuria jamesae*																																				
*Neoperla coosa*																																				
*Acroneuria filicis*																																				
*Perlesta shawnee*																																				
*Helopicus subvarians*	no adults were collected																																			
*Pteronarcys* spp.	no adults were collected																																			
